# Dominant Carbapenemase-Encoding Plasmids in Clinical Enterobacterales Isolates and Hypervirulent *Klebsiella pneumoniae*, Singapore

**DOI:** 10.3201/eid2808.212542

**Published:** 2022-08

**Authors:** Melvin Yong, Yahua Chen, Guodong Oo, Kai Chirng Chang, Wilson H.W. Chu, Jeanette Teo, Indumathi Venkatachalam, Natascha May Thevasagayam, Prakki S. Rama Sridatta, Vanessa Koh, Andrés E. Marcoleta, Hanrong Chen, Niranjan Nagarajan, Marimuthu Kalisvar, Oon Tek Ng, Yunn-Hwen Gan

**Affiliations:** National University of Singapore, Singapore (M. Yong, Y. Chen, G. Oo, K.C. Chang, W.H.W. Chu, N. Nagarajan, M. Kalisvar, Y.-H. Gan);; National University Hospital, Singapore (J. Teo);; Singapore General Hospital, Singapore (I. Venkatachalam);; National Centre for Infectious Diseases, Singapore (N.M. Thevasagayam, P.S.R. Sridatta, V. Koh, M. Kalisvar, O.T. Ng);; Tan Tock Seng Hospital, Singapore (V. Koh, M. Kalisvar, O.T. Ng);; Universidad de Chile, Santiago, Chile (A.E. Marcelota);; Genome Institute of Singapore, Singapore (H. Chen, N. Nagarajan);; Nanyang Technological University, Singapore (O.T. Ng)

**Keywords:** Antimicrobial resistance, bacteria, Enterobacterales, hypervirulent, capsule, KPC2, carbapenemase, *Klebsiella pneumoniae*, plasmid, conjugation, Singapore

## Abstract

Dissemination of carbapenemase-encoding plasmids by horizontal gene transfer in multidrug-resistant bacteria is the major driver of rising carbapenem-resistance, but the conjugative mechanics and evolution of clinically relevant plasmids are not yet clear. We performed whole-genome sequencing on 1,215 clinical Enterobacterales isolates collected in Singapore during 2010–2015. We identified 1,126 carbapenemase-encoding plasmids and discovered pKPC2 is becoming the dominant plasmid in Singapore, overtaking an earlier dominant plasmid, pNDM1. pKPC2 frequently conjugates with many Enterobacterales species, including hypervirulent *Klebsiella pneumoniae*, and maintains stability in vitro without selection pressure and minimal adaptive sequence changes. Furthermore, capsule and decreasing taxonomic relatedness between donor and recipient pairs are greater conjugation barriers for pNDM1 than pKPC2. The low fitness costs pKPC2 exerts in Enterobacterales species indicate previously undetected carriage selection in other ecological settings. The ease of conjugation and stability of pKPC2 in hypervirulent *K. pneumoniae* could fuel spread into the community.

The global rise of carbapenem-resistant Enterobacterales (CRE) infections is posing a grave challenge to hospital systems worldwide (*1*). Carbapenemase genes usually are located on plasmids that can transmit vertically along clonal lineages and horizontally between different strains and species (*2*). However, the principles governing the transmission of carbapenemase-encoding plasmids in clinically relevant settings are complex and dynamic. Plasmid properties, donor, recipient, and ecologic factors all affect transmission (*3*,*4*).

Previously, we found a 71,861-bp pKPC2 plasmid, pKPC2_sg1 (GenBank accession no. MN542377), in all 18 carbapenem-resistant hypervirulent *Klebsiella pneumoniae* isolates available in the Carbapenemase-Producing Enterobacteriaceae in Singapore (CaPES) collection (*5*,*6*). (Enterobacteriaceae is the former name of Enterobacterales.) The plasmid sequence was stable and unchanged after moving into different bacterial hosts or when maintained in human hosts for >200 days. This discovery prompted questions about the extent of pKPC2_sg1 dominance in clinical settings in Singapore, and its transmissibility and stability in hypervirulent *K. pneumoniae*. Using >1,000 CRE isolates collected from the 6 public hospitals in Singapore during 2010–2015, a subset of which was previously described (*6*), we examined the distribution of different carbapenem-encoding plasmids to investigate the dynamics and dominance of pKPC2. 

## Materials and Methods

### Bacterial Strains, Growth Conditions, and Plasmids

We analyzed 1,215 CRE isolates for carbapenemase plasmids distribution (Appendix 1 Table). We have included information on modified and unmodified plasmids, bacterial mutant generation and the Enterobacterales strains (Appendix 2). Unless otherwise specified, we grew strains on Lennox L Agar lysogeny agar (LA) (Invitrogen-ThermoFisher, https://www.thermofisher.com) at 37°C overnight before the assays. 

### Whole-Genome Sequencing 

We performed whole-genome sequencing (WGS) by using the MiSeq platform (Illumina, https://www.illumina.com) and the GridION X5 system (Oxford Nanopore Technologies, https://nanoporetech.com). To assemble genomes, we used SPAdes Genome Assembler version 3.11.1 (*7*) and Unicycler version 0.4.8 (*8*). For bacterial species assignment, we performed multilocus sequence typing (MLST) by using Bacterial Isolate Genome Sequence Database version 2.8 (*9*) or the Center for Genomic Epidemiology Bacterial Analysis Pipeline (https://www.genomicepidemiology.org) and Kraken (*10*). We identified antimicrobial resistance genes in CRE isolates by using Abricate version 1.0.1 (*11*) and the National Center for Biotechnology Information (NCBI) Bacterial Antimicrobial Resistance Reference Gene Database (*12*). We identified virulence genes by using the Virulence Factor Database (*13*) (Appendix 2).

### Plasmid Annotation and Analysis

We analyzed the pKPC2 sequence (GenBank accession no. MN542377) by using GeneMarkS (*14*) to acquire a list of predicted protein sequences and subjected sequences to blastp (*14*). We used blastp results to annotate genes on the plasmid, which we drew by using the BLAST Ring Image Generator (*15*). We also analyzed the plasmid sequence by using PlasmidFinder version 2.1 (*16*,*17*).

### Replicon Analysis

We used *Eco*RI and *Bam*HI restriction enzymes to double digest pKPC2 DNA for 1 hour at 37°C, then ligated the fragments to the pR6K plasmid by using T4 DNA Ligase (Promega, https://www.promega.com). We then used *Escherichia coli* Stellar HST08 Competent Cells (TaKaRa Bio, Inc., http://www.takara-bio.com) to introduce fragments through heat shock, and selected the transformants on LA with kanamycin (50 µg/mL). However, pR6K cannot replicate in HST08 cells because the R6K replicon protein must be provided in trans via lambda *pir*. Only pR6K with ligated fragments carrying a functional replicon can replicate. 

We harvested plasmids from the selected clones and submitted these to 1st BASE (https://base-asia.com) for Sanger sequencing to determine the inserts. We performed phylogenetic analysis on the identified *trfA* replicon by using ClustalW (https://www.clustal.org) and the maximum-likelihood method in MEGA-X version 10.2.6 (*18*).

### Bacterial Growth Assay

We streaked bacterial strains on LA containing antimicrobial drugs for various plasmids: 256 µg/mL erythromycin for pKPC2; 0.5 µg/mL meropenem for pNDM1; and 50 µg/mL kanamycin for pKPC2*^KmR^* or pNDM1*^KmR^*. We incubated plates at 37°C overnight, then inoculated colonies into Lennox L Broth Base lysogeny broth (LB; Invitrogen-ThermoFisher) containing the same antimicrobial drugs and placed in a shaking incubator set at 37°C and 150 rpm overnight. We measured the optical density at 600 nm (optical density 600) of overnight bacterial culture and recorded the reading before diluting it to 0.001. We added 200 µL of diluted cultures to a 96-well plate and placed these on a Synergy H1 plate reader (BioTek, https://www.biotek.com) at 37°C. We measured absorbance at optical density 600 hourly for 24 hours.

### Conjugation Experiments

We performed conjugation on 0.22 µm Cellulose Nitrate Filter (Sartorius, https://www.sartorius.com) nitrocellulose membranes using a 1:1 ratio of donor to recipient strains on LA. We measured plasmid transfer kinetics from *E. coli* MG1655 at various timepoints up to 4 hours at 37°C. We selected recipient strains on LA; *E. coli* SLC-568 with 50 µg/mL kanamycin or *K. pneumoniae* SGH10 with 40 µg/mL fosfomycin. We used the same antimicrobial drugs to select transconjugants on LA plus 256 µg/mL erythromycin for pKPC2 or 0.5 µg/mL meropenem for pNDM1. For conjugation assays of pKPC2*^KmR^* and pNDM1*^KmR^*, recipients carried pACYC184*^CmR^* for selection. We selected transconjugants on LA with 50 µg/mL chloramphenicol and 50 µg/mL kanamycin and selected recipients without kanamycin. For conjugation into hypervirulent *K. pneumoniae* recipients, we replaced chloramphenicol with 40 µg/mL fosfomycin. We measured conjugation frequency by dividing the number of transconjugants by the number of recipients.

### Plasmid Stability Assessment

We cultured strains in LB and 50 µg/mL kanamycin overnight, then subcultured every day by inoculating 4.88 µL of the culture into 5 mL of antimicrobial-free LB, as described (*19*). At generations 0, 30, 60, and 90, we serially diluted bacterial cultures and plated on LA with and without 50 µg/mL kanamycin. We further subcultured selected bacterial strains to 300 generations and plated at generation 100, 200, and 300. We calculated plasmid stability as the number of antimicrobial-resistant bacteria per total bacterial count. 

To test for plasmid incompatibility, we measured the stability of pKPC2*^KmR^* in *E. coli* MG1655 harboring both pKPC2*^KmR^* and pRK2-AraE as described (*20*), except we first grew the strain in LB with both 35 µg/mL gentamicin and 50 µg/mL kanamycin before subculturing for 100 generations in LB with 35 µg/mL gentamicin to select for pRK2-AraE. At every 10th generation, we plated the cultures on LA with 35 µg/mL gentamicin, and LA with 35 µg/mL gentamicin and 50 µg/mL kanamycin.

### Regression Analysis

To study the effect of taxonomic relatedness on pKPC2 and pNDM1 conjugation frequencies, we applied a survival-analysis approach (*21*). We modeled the donor-recipient pair as a random effect to account for unobserved heterogeneity specific to each pair (Appendix 2).

## Results

### Dominant Carbapenemase-Encoding Plasmid 

**Figure 1:**
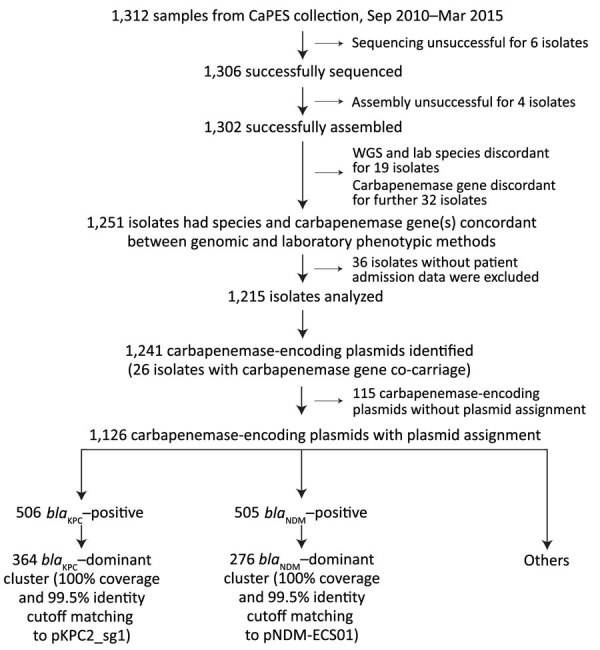
Flowchart of steps used for identifying dominant carbapenemase-encoding plasmids in clinical Enterobacterales isolates and hypervirulent *Klebsiella pneumoniae*, Singapore. We collected 1,312 samples available in the CaPES collection and analyzed 1,215 whole-genome sequenced samples. We identified 2 dominant clusters with large numbers of carbapenemase-encoding plasmids; the *bla*_KPC_*–*dominant cluster comprised pKPC2 plasmids and the *bla*_NDM_–dominant cluster pNDM1 plasmids. CaPES, Carbapenemase-Producing Enterobacteriaceae in Singapore (CaPES) (Enterobacteriaceae is the former name of Enterobacterales); WGS, whole-genome sequencing.

**Figure 2:**
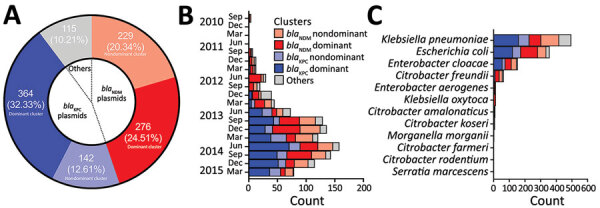
Percentage and distribution of dominant carbapenemase-encoding plasmids in clinical Enterobacterales isolates and hypervirulent *Klebsiella pneumoniae*, Singapore. A) Percentage distribution of the total carbapenemase-encoding plasmids identified. The *bla*_KPC_ dominant cluster refers to those harboring pKPC2 plasmid; *bla*_NDM_ dominant cluster refers to those harboring pNDM1 plasmid. Others indicate carbapenemase-encoding plasmids that do not carry *bla*_KPC_ or *bla*_NDM_. B, C) Distribution of carbapenemase-encoding plasmids identified among Carbapenemase-Producing Enterobacteriaceae in Singapore (CaPES) (Enterobacteriaceae is the former name of Enterobacterales) samples collected during September 2010–March 2015 (B) and among Enterobacterales isolates (C). Nondominant cluster refers to other plasmids carrying *bla*_KPC_ or *bla*_NDM_. We found that pKPC2 was the most dominant carbapenemase-encoding plasmid in Singapore during 2010–2015.

From 1,312 CRE isolates (817 unique patients) submitted during September 2010–April 2015 as part of mandatory reporting to the National Public Health Laboratory, we successfully cultured, performed WGS on, and assembled genomes for 1,302 (99.2%) isolates. Of those, 1,251 (96.1%) identified bacterial species and carbapenemase genes were concordant with laboratory data (MIC >1 mg/L or disc diffusion zone diameter <23 mm for imipenem and meropenem) (*6*). We excluded 36 isolates because patient or date of culture information was missing; thus, we analyzed 1,215 (93.3%) isolates (Figure 1; Appendix 1). We successfully identified 1,126 carbapenemase-encoding plasmids with assignments from the 1,215 isolates. We found 2 dominant carbapenemase-encoding plasmids: *bla*_KPC_ (n = 506; 44.94%) and *bla*_NDM_ (n = 505; 44.85%) (Figure 2, panel A). Among the 506 *bla*_KPC_ plasmids, 364 (32.33%) were pKPC2 plasmids, which we termed the *bla*_KPC_-dominant cluster. Among the 505 *bla*_NDM_ plasmids, 276 (24.51%) were pNDM1 plasmids, which we termed the *bla*_NDM_-dominant cluster. Nondominant plasmids included other *bla*_KPC_ or *bla*_NDM_ plasmids that did not fall into the pKPC2 or pNDM1 dominant clusters.

**Figure 3:**
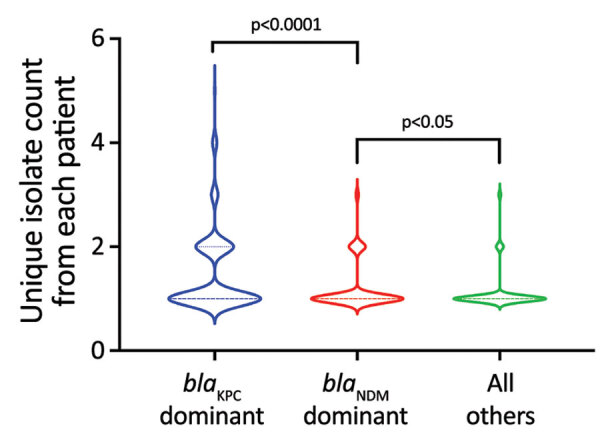
Violin plots showing the unique isolate counts from each patient in a study of dominant carbapenemase-encoding plasmids in clinical Enterobacterales isolates and hypervirulent *Klebsiella pneumoniae*, Singapore. Unique isolates were defined as different species or different sequence types from same species. We separated unique isolates into 3 groups: *bla*_KPC_ dominant (n = 196), *bla*_NDM_ dominant (n = 203), and all others (n = 504), which included *bla*_KPC_ nondominant, *bla*_NDM_ nondominant, and others. Brackets indicate p values for nonparametric Mann-Whitney tests between groups.

During 2010–2012, pNDM1 was predominant but pKPC2 subsequently caught up during 2013–2015 (Figure 2, panel B; Appendix 2 Figure 1, panel A). Those plasmids were largely found in 3 species: *K. pneumoniae* (43.96%), *E. coli* (31.71%), and *Enterobacter cloacae* (13.68%) (Figure 2, panel C). Bacterial sequence type (ST) distribution among *bla*_KPC_-positive and *bla*_NDM_-positive isolates showed that both *bla*_KPC_ and *bla*_NDM_ plasmids were widely distributed across numerous STs, particularly in *K. pneumoniae* (Appendix 2 Figure 1, panel B), indicating that widespread distribution is unlikely due to selective clonal expansion events. The *bla*_KPC_-dominant cluster also had more unique isolates than the other clusters, suggesting wider *bla*_KPC_ transmission (Figure 3).

### Evolution of pKPC2 Features 

**Figure 4:**
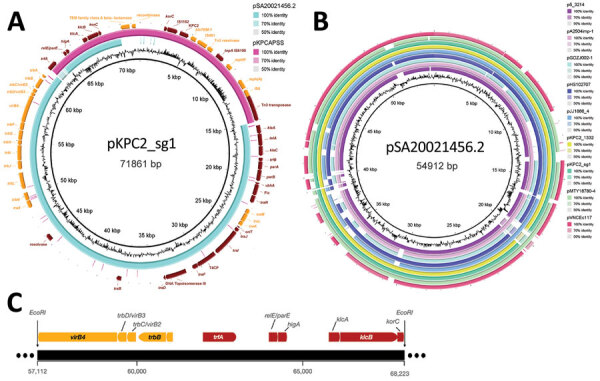
Annotated plasmid maps of the dominant carbapenemase-encoding plasmid in clinical Enterobacterales isolates and hypervirulent *Klebsiella pneumoniae*, Singapore. A) Annotated plasmid map of pKPC2_sg1 (GenBank accession no. MN542377), including the complete conjugative machinery (*oriT*, relaxase, T4CP, and T4SS) and the resistance genes, *bla*_KPC-2_, *bla*_TEM-1_, *mph*(A), and TEM family class A β-lactamase (TEM-1). The region of pKPC2_sg1 encoding the resistance genes was found in another plasmid called pKPCAPSS (GenBank accession no. KP008371), but the region encoding the conjugative machinery was highly similar to the sequence of pSA20021456.2 (GenBank accession no. CP030221). B) Plasmid alignment map showing other environmental or clinical plasmids with similar backbone, the pSA20021456.2 backbone, as pKPC2. C) Graphical representation of *Eco*RI*/Bam*HI digested pKPC2 region containing replicon.

Annotated features on the pKPC2 plasmid map show conjugative genes from the *tra* and *trb* operons and complete conjugative machinery (Figure 4, panel A). A comparison against the GenBank database for similar plasmids revealed pKPC2 is a hybrid of pSA20021456.2-like plasmids (GenBank accession no. CP030221), with 74% coverage and 99.60% identity, and pKPCAPSS-like plasmids (GenBank accession no. KP008371), with 34% coverage and 99.99% identity (Figure 4, panel A). The conjugative and plasmid maintenance genes in pKPC2 are encoded in the pSA20021456.2-like backbone, which also is found in several other plasmids carried by environmental or clinical isolates (Figure 4, panel B). The region with resistance genes matches part of pKPCAPSS, which might have originated from Southeast Asia (*22*). Using PlasmidFinder 2.1 (*16*), we were unable to find any replicon on pKPC2. To determine the potential origin of replication (oriV), we used restriction enzyme digestion to identify the gene fragment in pKPC2 capable of replication (*23*,*24*). We cloned the fragments into the lambda *pir*–dependent vector of pR6K. We successfully selected *E. coli* colonies with pR6K containing an 11,111-bp fragment with the *trfA* gene (Figure 4, panel C), which is the prototypical protein essential for replication of incompatibility group P (IncP) plasmids with *oriV* consisting of 5 17-bp tandem repeats (*25*). We detected 9 similar, but not identical, 17-bp tandem repeats immediately downstream of *trfA* (Appendix 2 Figure 2). We cloned the *trfA* and oriV region into pR6K and were able to successfully transform and replicate this region in *E. coli*, demonstrating that the *trfA* and *oriV* region is the minimal sequence required for replication (Appendix 2 Figure 3). 

**Figure 5:**
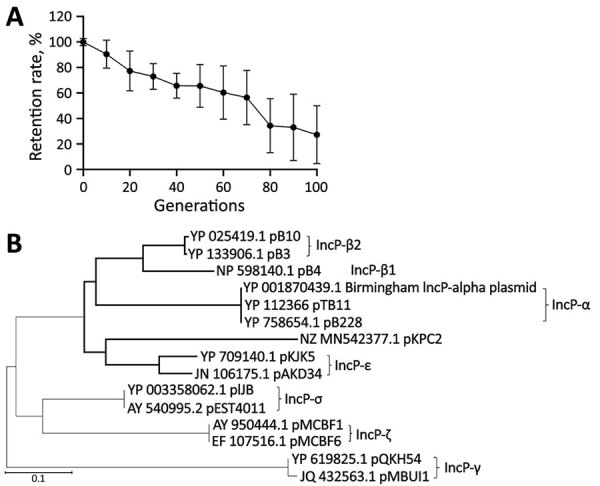
Plasmid incompatibility group analysis of pKPC2 carbapenemase-encoding plasmid in clinical Enterobacterales isolates and hypervirulent *Klebsiella pneumoniae*, Singapore. A) Stability of pKPC2*^KmR^* in *Escherichia coli* MG1655 harboring pRK2-AraE plasmid from IncP incompatibility group. Symbols indicate means and error bars indicate SDs from 3 independent experiments. B) Phylogenetic comparison of pKPC2 *trfA* replicon with other IncP plasmids among isolates. Scale bar indicates nucleotide substitutions per site.

To further examine whether the *trfA* replicon in pKPC2 belongs to the IncP family, we measured the plasmid stability of pKPC2 in presence of another IncP plasmid, pRK2. Plasmids that belong to the same incompatibility group cannot coexist stably in the same host because they have similar replicons (*26*). In *E. coli* MG1655 harboring both plasmids, pKPC2 was gradually lost when pRK2 was under selection (Figure 5, panel A). Moreover, phylogenetic analysis revealed that pKPC2’s *trfA* is related to the IncP family, but it does not belong to any existing subgroup and is more closely related to the IncP-ε subgroup, with some divergence (Figure 5, panel B). Analysis of pKPC2’s conjugative *tra* and *trb* operons also revealed the gene arrangement typical in IncP *tra1* and *tra2* cores (Appendix 2 Figure 4) (*27*).

### Stability and Genetic Adaptation of pKPC2 In Vitro

**Figure 6:**
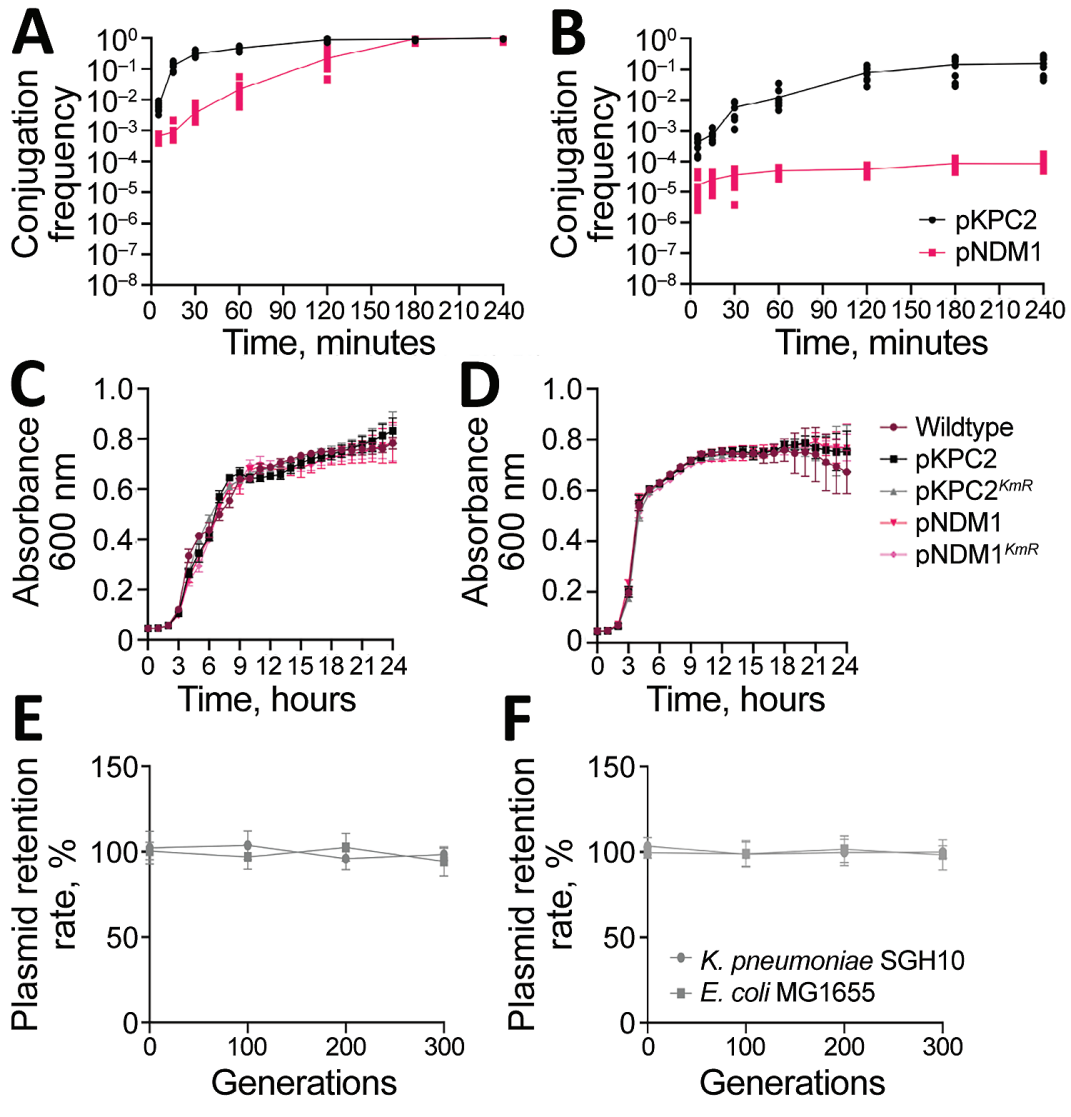
Characterization of pKPC2 carbapenemase-encoding plasmid in clinical Enterobacterales isolates and hypervirulent *Klebsiella pneumoniae*, Singapore. A, B) Conjugation kinetics of pKPC2 and pNDM1 from *Escherichia coli* MG1655 (donor) to *E. coli* SLC-568 (recipient) (A) or to *K. pneumoniae* SGH10 (recipient) (B). The donor-recipient pairs were mixed in 1:1 ratio and conjugated for 4 hours at 37°C using filter matings. The number of transconjugant and recipient pairs were enumerated by plating. Results from 3 independent experiments were plotted as the conjugation frequency (transconjugant/recipient) over time (minutes). Error bars indicate SDs from 3 independent experiments. C, D) Representative growth curve of *E. coli* MG1655 (C) or *K. pneumoniae* SGH10 (D) with or without plasmids pKPC2, pKPC2*^KmR^*, pNDM1, pNDM1*^KmR^* grown in LB media at 37°C for 24 h. E) Stability of pKPC2*^KmR^* (E) and pNDM1*^KmR^* (F) in *K. pneumoniae* SGH10 and *E. coli* MG1655 grown in LB up to generation 300. Symbols indicate means and error bars indicate SDs from 3 independent experiments. LB, lysogeny broth.

pKPC2 exhibited faster conjugation kinetics, reaching nearly 10^0^ after 2–3 hours, than did pNDM1 (GenBank accession no. JADPQD010000004), which took 3–4 hours to reach 10^0^ (Figure 6, panel A). With hypervirulent *K. pneumoniae* SGH10 as the recipient, the conjugation frequency remained higher for pKPC2 than for pNDM1 (Figure 6, panel B).

To determine whether those plasmids exert any fitness cost on host strains, we measured the growth rate of host strains in presence or absence of the plasmids. We included plasmids tagged with kanamycin resistance, pKPC2*^KmR^* and pNDM1*^KmR^*, because they were used for subsequent experiments with kanamycin as a robust selection marker. We found no significant difference in growth rate for *E. coli* MG1655 or *K. pneumoniae* SGH10 (Figure 6, panels C, D). To simulate a nutrient-poor condition, we tested growth rates in minimal media, which also showed no significant growth differences (Appendix 2 Figure 5). Furthermore, both plasmids remained stable for up to 300 generations without selection pressure (Figure 6, panels E, F). We compared the sequences of the 9 pKPC2*^KmR^* plasmids from the 300th generation (pKPC2*^KmR^*_Gen300) *K. pneumoniae* SGH10 isolates to the original pKPC2*^KmR^* plasmid using in vitro plasmid evolution experiments and noted no major changes in the plasmid sequence (Appendix 2 Figure 6). Among the nine 300th-generation plasmids, 6 had 2 or 4 nucleotide mismatches on β-lactamase genes. However, sequence comparison of the pKPC2 and pKPC2*^KmR^* used in this study to the pKPC2_sg1 from the clinical isolate *K. pneumoniae* ENT494 (GenBank accession no. MN542377) shows the same nucleotide polymorphism in the same genes (Appendix 2 Figure 7), indicating that these are likely the only bona fide evolved adaptations of the plasmid. Because host bacteria can also evolve to adapt to plasmid carriage (*28*), we compared the genomic sequences of nine 300th-generation *K. pneumoniae* SGH10 isolates carrying pKPC2 and nine 300th-generation isolates without pKPC2. We hypothesized that host adaptation would lead to an increased number of nonsynonymous mutations in the strains carrying the plasmid versus the plasmid-null strains, leading to changes in protein function. However, our results indicated similar numbers of synonymous, nonsynonymous, and total nucleotide polymorphism differences in both groups.

### pKPC2 Conjugation Frequency and Stability in Enterobacterales Species

We hypothesize that the predominance of pKPC2 in our clinical isolates is due to its high conjugation frequency to different Enterobacterales species. The conjugation frequency of pKPC2*^KmR^* from MG1655 to other *E. coli* or *E. cloacae* recipient strains were remarkably high, ranging from 10^−1^ to 10^0^ (Appendix 2 Figure 8, panel A). We observed the same conjugation frequency for several clinical *Klebsiella* strains, such as *K. pneumoniae* NUH29, *K. quasipneumoniae* TTSH4, *K. oxytoca* 8071169380, and *K. variicola* NUH59. However, some *Klebsiella* recipient strains exhibited lower conjugation frequency, in the 10^−3^ to 10^−1^ range. For pNDM1*^KmR^*, the conjugation frequency was ≈10–100-fold lower than for pKPC2*^KmR^* for most pairs. When we used *K. pneumoniae* SGH10 as the donor to the same panel of Enterobacterales recipients, the conjugation frequency of both plasmids was 10–100-fold lower than when *E. coli* MG1655 was the donor (Appendix 2 Figure 8, panel B). We then swapped the donor-recipient pairs by using the panel of Enterobacterales strains as donors and *K. pneumoniae* SGH10 as the recipient (Appendix 2 Figure 8, panel C). Overall, the conjugation frequency for pKPC2*^KmR^* remained higher than the frequency for pNDM1*^KmR^* in most donor-recipient pairs. However, the conjugation frequency of the swapped donor-recipient pairs was not the same as the original pairs, indicating the effects of donor and recipient factors. Both plasmids within the Enterobacterales strains were stable for up to 90 generations, except for *E. coli* UTI89, which failed to retain the pKPC2*^KmR^* plasmid (Appendix 2 Figure 8, panels D, E). These results align with clinical data showing the persistence of the pKPC2 plasmid over several months in patients without antimicrobial drug exposure (*5*).

### Conjugation Frequency and Stability of pKPC2 in Hypervirulent *K. pneumoniae*

Because pKPC2 was previously found in 18 local clinical hypervirulent *K. pneumoniae* isolates of K1, K2, and K20 capsular serotypes (*5*), we hypothesize that the plasmid does not face constraints in transmission to hypervirulent *K. pneumoniae*. Those isolates were loosely defined as hypervirulent *K. pneumoniae* based on occurrence of >2 virulence genes, such as *iro* and *rmpA* (Appendix 2 Table 1). Indeed, we observed high conjugation frequency for K1 strains (Appendix 2 Figure 9, panel A). On the other hand, K2 and K5 strains exhibited heterogeneity in their plasmid acceptance. However, plasmid conjugation success was independent of capsular types because we observed low conjugation frequency in 2 STs, K2/ST2039 and K5/ST60, whereas other STs of the same capsular type exhibited markedly higher conjugation frequency. Compared with pNDM1*^KmR^* (Appendix 2 Figure 9, panel B), the conjugation frequency of pKPC2*^KmR^* was ≈10–100-fold higher. In fact, K2/ST2039 and K5/ST60 strains were low conjugators for both plasmids. Despite the low conjugation frequency, the plasmids maintained stability over 90 generations (Appendix 2 Figure 10).

### Effects of Taxonomic Factors on pNDM1 Conjugation 

To examine the influence of taxonomic factors on pKPC2 and pNDM1 conjugation frequencies, we performed statistical analyses on available datasets (Appendix 2 Figures 8, 9) by using a survival-analysis approach (*21*). Comparing the baseline conjugation frequency between the same strain, we noted a statistically significant decrease in pKPC2 transfer between the same species (24.0-fold) or same genus (10.2-fold) but no statistically significant decrease between different genera (Table). On the other hand, we noted a statistically significant decrease in pNDM1 transfer between the same species (36.3-fold), same genus (123.0-fold), and different genera (87.1-fold). These results suggest that taxonomic factors have a higher influence on pNDM1 than pKPC2, which is especially notable for transfer between the same genus or different genera.

**Table:** Regression coefficients for pKPC2 and pNDM1 plasmid conjugation frequencies between donor-recipient pairs in a study of carbapenemase-encoding plasmids in clinical Enterobacterales isolates and hypervirulent *Klebsiella pneumoniae*, Singapore*

Taxonomic relatedness (model parameter)	pKPC2			pNDM1	
	Mean	Bootstrap 95% CI		Mean	Bootstrap 95% CI
Same strain (*β*_0_)	−1.73	−1.824 to −1.610		−2.31	−2.405 to −2.212
Same species (*β*_1_)	−1.38	−1.599 to −1.147		−1.56	−1.771 to −1.317
Same genus (*β*_2_)	−1.01	−1.162 to −0.866		−2.09	−2.258 to −1.907
Different genus (*β*_3_)	0.04	−0.1165 to 0.1988		−1.94	−2.086 to −1.808

*log base 10 conjugation frequencies and their bootstrap 95% CI as described in for the regression equation and fitting procedure are shown.

### Effect of Bacterial Capsule on Plasmid Conjugation

We examined a panel of isogenic deletion mutants of *K. pneumoniae* SGH10 as recipients that could affect donor-recipient pair mating dynamics. Conjugation frequency was enhanced in Δ*rmpA* and ΔICEKp10 recipients, but the greatest impediment to plasmid conjugation was the capsule (Appendix 2 Figure 9, panel C). The Δ*wcaJ* recipient exhibited conjugation efficiency approaching 10^0^ for both plasmids (Appendix 2 Figure 9, panel D). Similarly, capsule absence increased the conjugation frequency of both plasmids from *E. coli* MG1655 to capsule-null mutants of the low conjugating hypervirulent *K. pneumoniae* isolates (Appendix 2 Figure 11). However, the increases in conjugation frequency of pNDM1*^KmR^* in Δ*wcaJ* suggests that capsule is not as much of a barrier to pKPC2 as it is to pNDM1.

## Discussion

The spread of carbapenemase-encoding plasmids via horizontal gene transfer poses a major challenge to treatment against multidrug-resistant gram-negative bacteria because carbapenems are often antimicrobial agents of last resort. However, the dynamics and factors enabling the spread of these clinically significant plasmids have not been well studied. Previously, we found that pKPC2 is the only carbapenemase-encoding plasmid harbored by all the carbapenemase-resistant hypervirulent *K. pneumoniae* identified (*5*). Hypervirulent *K. pneumoniae* can cause *Klebsiella-*induced liver abscess, a community-acquired infection endemic in Asia-Pacific regions (*29*); the K1/ST23 lineage is predominantly responsible and causes 80% of these abscesses (*30*). Hypervirulent *K. pneumoniae* evolved through separate lineages from classical strains that typically cause multidrug-resistant nosocomial infections (*30*). Because hypervirulent *K. pneumoniae* is thought to be less receptive to horizontal gene transfer, pKPC2 in these strains could indicate that this plasmid has high transmission potential. 

Our results showed that pKPC2 was the most prevalent carbapenemase-encoding plasmid among the clinical Enterobacterales isolates in CaPES. These plasmids are largely found in *K. pneumoniae*, *E. coli*, and *E. cloacae*, which also were the most prevalent carbapenemase-encoding plasmid-harboring species reported in other surveillance studies (*31*,*32*), showing that those are major reservoirs. Although KPC-2 has been documented on diverse plasmids and is known to undergo frequent recombination events (*33*), we uncovered a single plasmid that moves as a discrete and intact unit among diverse strains and species. One limitation of our epidemiologic study is that we do not yet know whether the same trend in plasmid transfer persisted after 2015.

Several factors revealed by our in vitro data potentially explain the high prevalence and dominance of pKPC2 in clinical isolates. First, pKPC2 conjugates with fast kinetics and has high transmissibility among various host-recipient pairs. Although taxonomic relatedness is known to affect conjugation frequency (*21*), pNDM1 is more strongly affected by this relatedness than pKPC2, especially for transfer within same and other genera. This finding likely accounts for the success of pKPC2 as the dominant carbapenemase-encoding plasmid among Enterobacterales clinical isolates. Second, pKPC2 has low fitness costs and is highly adapted to host species. The persistence of plasmids in bacterial populations over an extended period has long been regarded as an evolutionary dilemma (*34*). Although compensatory mechanisms could account for plasmid persistence within a community with a high conjugation rate, offsetting the disadvantage incurred by high fitness cost in the absence of selection pressure (*35*), another study reported that the key factor for the persistence of the pOXA48_K8 plasmid is its low fitness costs across many clinical Enterobacteriaceae hosts in the gut, rather than its high conjugation frequency (*36*,*37*). We found that pKPC2 imposes low fitness cost and had high conjugation frequency across several Enterobacterales isolates and a remarkable retention rate, even in low conjugating strains. pKPC2 exhibited no mutations after in vitro evolution experiments and almost no changes compared with original clinical isolates. 

We noted that both the conjugative machinery and plasmid maintenance genes in pKPC2 are encoded by the pSA20021456.2-like backbone. Several plasmids with a similar backbone have been described (Figure 4, panel B), including the multidrug-resistant pHS102707 and the pJJ1886_4 plasmids found in clinical *E. coli* strains (*38*,*39*). This finding raises the concern that plasmids with this backbone might have similar dissemination potential or be able to recombine with plasmid fragments bearing multidrug-resistant genes and a suitable oriV to become dominant under antimicrobial drug selection pressure. Although we might never know the origins and the evolutionary steps taken by pKPC2, one clue is its phylogenetic relatedness to IncP-ε plasmids, which have been observed to be vectors in the spread of antimicrobial drug resistance in agricultural systems (*40*).

The high transmissibility of pKPC2 was also seen in hypervirulent *K. pneumoniae* clinical isolates. Hypervirulent *K. pneumoniae* is thought to face constraints in horizontal gene transfer, and its low gene content diversity further supported the idea that the thick capsular polysaccharide is a barrier to transfer (*41*). Reports of ∆*wcaJ* in 4 different strains of *K. pneumoniae* showed an 8–20-fold increase in plasmid conjugation over 1 hour (*42*). Capsule deletion increased conjugation frequency by 10–100-fold in pNDM1 compared with pKPC2. This increase shows the capsule is more of a hindrance to pNDM1 than to pKPC2, suggesting that pKPC2 has a competitive advantage over pNDM1 in its transmission to encapsulated strains. This finding might explain why pKPC2 is the only carbapenemase-encoding plasmid among all the hypervirulent/carbapenem-resistant *K. pneumoniae* isolates we discovered (*5*). The high transmissibility of pKPC2 to the antimicrobial-sensitive, community-acquired hypervirulent *K. pneumoniae* strains suggests that pKPC2 or its predecessors might have undergone carriage selection for high transmissibility and persistence in isolates from ecologic settings that harbor similar features to hypervirulent *K. pneumoniae*. Although our mechanistic studies of plasmid transmission are limited to in vitro experiments, these studies provide insights and potential explanations on the pattern of transmission observed clinically.

In summary, this study underscores the need to track the spread and dominance of clinically relevant carbapenemase-encoding plasmids in healthcare settings and examine transmission characteristics. Our findings reveal increasing dominance of pKPC2 over other carbapenemase-encoding plasmids during a 5-year period. pKPC2 appears to be a highly adapted hybrid plasmid exhibiting increased transmissibility and persistence among Enterobacterales and hypervirulent *K. pneumoniae* strains. These highly evolved and adapted plasmids act as agents that move easily between various hosts and exert negligible fitness costs, facilitating their long-term carriage even without selection pressure. We propose that the pKPC2 plasmid has already undergone carriage adaptation and been in circulation for some time. Insights gained on the transmission potential of pKPC2 and other similarly evolved plasmids could translate into better infection prevention measures or improved surveillance. 

Appendix 1 Isolates used to investigate dominant carbapenemase-encoding plasmids in clinical Enterobacterales isolates and hypervirulent *Klebsiella pneumoniae*, Singapore.

Appendix 2 Additional information on dominant carbapenemase-encoding plasmids in clinical Enterobacterales isolates and hypervirulent *Klebsiella pneumoniae*, Singapore.

## References

[1] World Health Organization. Global priority list of antibiotic-resistant bacteria to guide research, discovery, and development of new antibiotics. Geneva: The Organization; 2017.

[2] Yang X, Dong N, Chan EW, Zhang R, Chen S. Carbapenem resistance-encoding and virulence-encoding conjugative plasmids in *Klebsiella pneumoniae.* Trends Microbiol. 2021;29:65–83. 10.1016/j.tim.2020.04.012 32448764

[3] Benz F, Huisman JS, Bakkeren E, Herter JA, Stadler T, Ackermann M, et al. Plasmid- and strain-specific factors drive variation in ESBL-plasmid spread in vitro and in vivo. ISME J. 2021;15:862–78. 10.1038/s41396-020-00819-4 33149210 PMC8026971

[4] Rodríguez-Beltrán J, DelaFuente J, León-Sampedro R, MacLean RC, San Millán Á. Beyond horizontal gene transfer: the role of plasmids in bacterial evolution. Nat Rev Microbiol. 2021;19:347–59. 10.1038/s41579-020-00497-1 33469168

[5] Chen Y, Marimuthu K, Teo J, Venkatachalam I, Cherng BPZ, De Wang L, et al. Acquisition of plasmid with carbapenem-resistance gene *bla*_KPC2_ in hypervirulent *Klebsiella pneumoniae*, Singapore. Emerg Infect Dis. 2020;26:549–59. 10.3201/eid2603.191230 32091354 PMC7045839

[6] Marimuthu K, Venkatachalam I, Khong WX, Koh TH, Cherng BPZ, Van La M, et al.; Carbapenemase-Producing Enterobacteriaceae in Singapore (CaPES) Study Group. Carbapenemase-Producing Enterobacteriaceae in Singapore (CaPES) Study Group. Clinical and molecular epidemiology of carbapenem-resistant Enterobacteriaceae among adult inpatients in Singapore. Clin Infect Dis. 2017;64(suppl_2):S68–75. 10.1093/cid/cix113 28475792

[7] Bankevich A, Nurk S, Antipov D, Gurevich AA, Dvorkin M, Kulikov AS, et al. SPAdes: a new genome assembly algorithm and its applications to single-cell sequencing. J Comput Biol. 2012;19:455–77. 10.1089/cmb.2012.0021 22506599 PMC3342519

[8] Wick RR, Judd LM, Gorrie CL, Holt KE. Unicycler: Resolving bacterial genome assemblies from short and long sequencing reads. PLOS Comput Biol. 2017;13:e1005595. 10.1371/journal.pcbi.1005595 28594827 PMC5481147

[9] Jolley KA, Maiden MC. BIGSdb: Scalable analysis of bacterial genome variation at the population level. BMC Bioinformatics. 2010;11:595. 10.1186/1471-2105-11-595 21143983 PMC3004885

[10] Wood DE, Salzberg SL. Kraken: ultrafast metagenomic sequence classification using exact alignments. Genome Biol. 2014;15:R46. 10.1186/gb-2014-15-3-r46 24580807 PMC4053813

[11] Seemann T. Abricate [cited 2021 Aug 5]. https://github.com/tseemann/abricate

[12] Feldgarden M, Brover V, Haft DH, Prasad AB, Slotta DJ, Tolstoy I, et al. Validating the AMRFinder tool and resistance gene database by using antimicrobial resistance genotype-phenotype correlations in a collection of isolates. Antimicrob Agents Chemother. 2019;63:e00483–19. 10.1128/AAC.00483-19 31427293 PMC6811410

[13] Chen L, Zheng D, Liu B, Yang J, Jin Q. VFDB 2016: hierarchical and refined dataset for big data analysis—10 years on. Nucleic Acids Res. 2016;44(D1):D694–7. 10.1093/nar/gkv1239 26578559 PMC4702877

[14] Besemer J, Lomsadze A, Borodovsky M. GeneMarkS: a self-training method for prediction of gene starts in microbial genomes. Implications for finding sequence motifs in regulatory regions. Nucleic Acids Res. 2001;29:2607–18. 10.1093/nar/29.12.2607 11410670 PMC55746

[15] Alikhan NF, Petty NK, Ben Zakour NL, Beatson SA. BLAST Ring Image Generator (BRIG): simple prokaryote genome comparisons. BMC Genomics. 2011;12:402. 10.1186/1471-2164-12-402 21824423 PMC3163573

[16] Carattoli A, Zankari E, García-Fernández A, Voldby Larsen M, Lund O, Villa L, et al. In silico detection and typing of plasmids using PlasmidFinder and plasmid multilocus sequence typing. Antimicrob Agents Chemother. 2014;58:3895–903. 10.1128/AAC.02412-14 24777092 PMC4068535

[17] Robertson J, Nash JHE. MOB-suite: software tools for clustering, reconstruction and typing of plasmids from draft assemblies. Microb Genom. 2018;4:4. 10.1099/mgen.0.000206 30052170 PMC6159552

[18] Kumar S, Stecher G, Li M, Knyaz C, Tamura K. MEGA X: Molecular Evolutionary Genetics Analysis across computing platforms. Mol Biol Evol. 2018;35:1547–9. 10.1093/molbev/msy096 29722887 PMC5967553

[19] De Gelder L, Ponciano JM, Joyce P, Top EM. Stability of a promiscuous plasmid in different hosts: no guarantee for a long-term relationship. Microbiology (Reading). 2007;153:452–63. 10.1099/mic.0.2006/001784-0 17259616

[20] Cook TB, Rand JM, Nurani W, Courtney DK, Liu SA, Pfleger BF. Genetic tools for reliable gene expression and recombineering in Pseudomonas putida. J Ind Microbiol Biotechnol. 2018;45:517–27. 10.1007/s10295-017-2001-5 29299733 PMC6161825

[21] Alderliesten JB, Duxbury SJN, Zwart MP, de Visser JAGM, Stegeman A, Fischer EAJ. Effect of donor-recipient relatedness on the plasmid conjugation frequency: a meta-analysis. BMC Microbiol. 2020;20:135. 10.1186/s12866-020-01825-4 32456625 PMC7249681

[22] Ageevets V, Sopova J, Lazareva I, Malakhova M, Ilina E, Kostryukova E, et al. Genetic environment of the *bla*_KPC-2_ Gene in a *Klebsiella pneumoniae* isolate that may have been imported to Russia from Southeast Asia. Antimicrob Agents Chemother. 2017;61:e01856–16. 10.1128/AAC.01856-16 27919902 PMC5278738

[23] Miki T, Easton AM, Rownd RH. Cloning of replication, incompatibility, and stability functions of R plasmid NR1. J Bacteriol. 1980;141:87–99. 10.1128/jb.141.1.87-99.1980 6243631 PMC293536

[24] Lovett MA, Helinski DR. Method for the isolation of the replication region of a bacterial replicon: construction of a mini-F’kn plasmid. J Bacteriol. 1976;127:982–7. 10.1128/jb.127.2.982-987.1976 783124 PMC233008

[25] Thomas CM, Smith CA. Incompatibility group P plasmids: genetics, evolution, and use in genetic manipulation. Annu Rev Microbiol. 1987;41:77–101. 10.1146/annurev.mi.41.100187.000453 3318684

[26] Novick RP. Plasmid incompatibility. Microbiol Rev. 1987;51:381–95. 10.1128/mr.51.4.381-395.1987 3325793 PMC373122

[27] Pansegrau W, Lanka E, Barth PT, Figurski DH, Guiney DG, Haas D, et al. Complete nucleotide sequence of Birmingham IncP alpha plasmids. Compilation and comparative analysis. J Mol Biol. 1994;239:623–63. 10.1006/jmbi.1994.1404 8014987

[28] Stalder T, Rogers LM, Renfrow C, Yano H, Smith Z, Top EM. Emerging patterns of plasmid-host coevolution that stabilize antibiotic resistance. Sci Rep. 2017;7:4853. 10.1038/s41598-017-04662-0 28687759 PMC5501780

[29] Lee CR, Lee JH, Park KS, Jeon JH, Kim YB, Cha CJ, et al. Antimicrobial resistance of hypervirulent *Klebsiella pneumoniae*: epidemiology, hypervirulence-associated determinants, and resistance mechanisms. Front Cell Infect Microbiol. 2017;7:483. 10.3389/fcimb.2017.00483 29209595 PMC5702448

[30] Lam MMC, Wyres KL, Duchêne S, Wick RR, Judd LM, Gan YH, et al. Population genomics of hypervirulent *Klebsiella pneumoniae* clonal-group 23 reveals early emergence and rapid global dissemination. Nat Commun. 2018;9:2703. 10.1038/s41467-018-05114-7 30006589 PMC6045662

[31] Sugawara Y, Hagiya H, Akeda Y, Aye MM, Myo Win HP, Sakamoto N, et al. Dissemination of carbapenemase-producing Enterobacteriaceae harbouring bla_NDM_ or bla_IMI_ in local market foods of Yangon, Myanmar. Sci Rep. 2019;9:14455. 10.1038/s41598-019-51002-5 31595007 PMC6783431

[32] Miltgen G, Cholley P, Martak D, Thouverez M, Seraphin P, Leclaire A, et al. Carbapenemase-producing Enterobacteriaceae circulating in the Reunion Island, a French territory in the Southwest Indian Ocean. Antimicrob Resist Infect Control. 2020;9:36. 10.1186/s13756-020-0703-3 32075697 PMC7031992

[33] David S, Cohen V, Reuter S, Sheppard AE, Giani T, Parkhill J, et al.; European Survey of Carbapenemase-Producing Enterobacteriaceae (EuSCAPE) Working Group; ESCMID Study Group for Epidemiological Markers (ESGEM). Integrated chromosomal and plasmid sequence analyses reveal diverse modes of carbapenemase gene spread among *Klebsiella pneumoniae.* Proc Natl Acad Sci U S A. 2020;117:25043–54. 10.1073/pnas.2003407117 32968015 PMC7587227

[34] Harrison E, Brockhurst MA. Plasmid-mediated horizontal gene transfer is a coevolutionary process. Trends Microbiol. 2012;20:262–7. 10.1016/j.tim.2012.04.003 22564249

[35] Lopatkin AJ, Meredith HR, Srimani JK, Pfeiffer C, Durrett R, You L. Persistence and reversal of plasmid-mediated antibiotic resistance. Nat Commun. 2017;8:1689. 10.1038/s41467-017-01532-1 29162798 PMC5698434

[36] Alonso-Del Valle A, León-Sampedro R, Rodríguez-Beltrán J, DelaFuente J, Hernández-García M, Ruiz-Garbajosa P, et al. Variability of plasmid fitness effects contributes to plasmid persistence in bacterial communities. Nat Commun. 2021;12:2653. 10.1038/s41467-021-22849-y 33976161 PMC8113577

[37] León-Sampedro R, DelaFuente J, Díaz-Agero C, Crellen T, Musicha P, Rodríguez-Beltrán J, et al.; R-GNOSIS WP5 Study Group. Pervasive transmission of a carbapenem resistance plasmid in the gut microbiota of hospitalized patients. Nat Microbiol. 2021;6:606–16. 10.1038/s41564-021-00879-y 33782584 PMC7610705

[38] Li G, Zhang Y, Bi D, Shen P, Ai F, Liu H, et al. First report of a clinical, multidrug-resistant *Enterobacteriaceae* isolate coharboring fosfomycin resistance gene *fosA3* and carbapenemase gene *bla*_KPC-2_ on the same transposon, Tn*1721.* Antimicrob Agents Chemother. 2015;59:338–43. 10.1128/AAC.03061-14 25367902 PMC4291370

[39] Andersen PS, Stegger M, Aziz M, Contente-Cuomo T, Gibbons HS, Keim P, et al. Complete genome sequence of the epidemic and highly virulent CTX-M-15–producing *H*30-Rx subclone of *Escherichia coli* ST131. Genome Announc. 2013;1:e00988–13. 10.1128/genomeA.00988-13 24309736 PMC3853059

[40] Heuer H, Binh CT, Jechalke S, Kopmann C, Zimmerling U, Krögerrecklenfort E, et al. IncP-1ε plasmids are important vectors of antibiotic resistance genes in agricultural systems: diversification driven by class 1 integron gene cassettes. Front Microbiol. 2012;3:2. 10.3389/fmicb.2012.00002 22279444 PMC3260659

[41] Wyres KL, Wick RR, Judd LM, Froumine R, Tokolyi A, Gorrie CL, et al. Distinct evolutionary dynamics of horizontal gene transfer in drug resistant and virulent clones of *Klebsiella pneumoniae.* PLoS Genet. 2019;15:e1008114. 10.1371/journal.pgen.1008114 30986243 PMC6483277

[42] Haudiquet M, Buffet A, Rendueles O, Rocha EPC. Interplay between the cell envelope and mobile genetic elements shapes gene flow in populations of the nosocomial pathogen *Klebsiella pneumoniae.* PLoS Biol. 2021;19:e3001276. 10.1371/journal.pbio.3001276 34228700 PMC8259999

