# Supplementary material for: Dominant Carbapenemase-Encoding Plasmids in Clinical Enterobacterales Isolates and Hypervirulent Klebsiella pneumoniae, Singapore
Source: Emerg Infect Dis. 2022 Aug;28(8):1578–88. doi: 10.3201/eid2808.212542 (PMC9328930; doi:10.3201/eid2808.212542)
Supplement: Appendix 2 — Additional information on dominant carbapenemase-encoding plasmids in clinical Enterobacterales isolates and hypervirulent Klebsiella pneumoniae, Singapore. [file 21-2542-Techapp-s2.pdf]

Article DOI: https://doi.org/10.3201/eid2808.212542

Dominant Carbapenemase-Encoding Plasmids in Clinical Enterobacterales Isolates and Hypervirulent *Klebsiella pneumoniae*, Singapore

Appendix 2

Supplementary Methods

Mutant Generation

To delete genes/regions in hypervirulent *Klebsiella pneumonia*e, ≈1,000-bp fragments upstream and downstream of the target were amplified with PCR (Appendix 2 Table 3) from genomic DNA templates with Taq polymerase and then assembled in a conditional suicide pR6KmobsacB vector by using NEBuilder HiFi DNA Assembly Master Mix (New England Biolabs). The vectors were then transformed into *Escherichia coli* S17–1λpir competent cells before being conjugated into hypervirulent *K. pneumoniae* strains. Single crossover transconjugants were selected on lysogeny agar with kanamycin + fosfomycin (50 µg/mL + 40 µg/mL) and subcultured in lysogeny broth containing 15% sucrose without NaCl. Kanamycin-sensitive, double crossovers were screened for successful deletion by PCR. For the generation of capsule null mutants in NUH66, TTSH25, and SGH07, genomes were first annotated with Prokka 1.13 (*1*) to locate the initial glycotransferase *wcaJ* gene. The *wcaJ* gene was deleted in each strain by using the same method.

Construction of Plasmids

Plasmids were introduced into bacterial strains by electroporation, chemical transformation, or conjugation (Appendix 2 Table 2). Plasmids were constructed by first amplifying individual fragments from various plasmids by using Q5 High-Fidelity DNA Polymerase (New England Biolabs, NEB) in PCR and then all the fragments were assembled by using NEBuilder HiFi DNA Assembly Cloning Kit (NEB). Plasmid mutants were generated

based on the Lambda Red system (*2*) with some modifications. First, a curable Lambda Red plasmid (pACYC-flp-Red-sacB) was generated by combining the enhanced Flp recombinase gene from pFLPe2 (*3*), the Lambda Red recombinase genes from pKD46 (*4*), and the *sac*B gene from pK18mobsacB (*5*) into pACYC184 (*6*). Electroporation-competent cells of *E. coli* MG1655 carrying pACYC-flp-Red-sacB and pKPC2 or pNDM1 were prepared in the presence of L-arabinose (0.2%). The competent cells were electroporated with PCR product of chloramphenicol resistance gene (CmR) or kanamycin resistance gene (KmR) with 40-nt extensions that are homologous to regions adjacent to the gene to be inactivated. CmR or KmR transformants were selected. Gene replacement was verified by colony PCR. The pACYC-flp-Red-sacB plasmid was cured from the strain by growing them in medium containing sucrose (15%).

Plasmid Genome Sequence Reference Database

Reference plasmid genome sequences for each carbapenemase gene allele were derived from 2 sources: NCBI RefSeq database (https://www.ncbi.nlm.nih.gov/refseq) or fully circularized plasmid genome sequences from hybrid assemblies based on Oxford Nanopore Technologies (ONT, https://nanoporetech.com). Complete plasmid sequences were downloaded from NCBI RefSeq database by using the search term "plasmid." The downloaded sequence data were further filtered to only include plasmid sequences with identifiers that met the keyword criteria "plasmid" AND "circular" NOT "gene" NOT "partial" NOT "incomplete" NOT "putative." Additionally, the dataset was filtered to remove exact duplicate sequences. All known alleles from ARG-ANNOT database (*7*) were downloaded and plasmid sequences were aligned against the carbapenemase gene allele sequences. Only plasmids that had 100% coverage and 100% identity with the carbapenemase gene allele were incorporated into the database (n = 396). Second, a set of fully circularized plasmid genome sequences (n = 542) extracted from ONT-based hybrid assemblies were added to the reference database using the same criteria for the specific carbapenemase gene allele presence. In total, the plasmid genome sequence reference database contained 938 complete plasmid sequences. Subsequently, a self-BLAST was performed and sequences that shared 99% identity, 99% query coverage, and 99% subject coverage were grouped into clusters.

Identification of Carbapenemase-Encoding Plasmids for Each Isolate

For each isolate, fully circularized CP sequence was identified from among the 542 hybrid assemblies described above, if available. For isolates that did not have a fully circularized carbapenemase-encoding plasmids sequence, plasmid identification was performed using the following steps: (i) PlasmidSeeker (v0.1; 2017–04–21) was run using the Illumina reads as input and with default parameters against the carbapenemase gene allele-specific reference database described above (n = 938) to obtain candidate plasmids; (ii) For each isolate, the carbapenemase gene-containing contig (carrying the carbapenemase gene with at least 99% identity and 90% gene coverage) was identified from the Illumina assembly; (iii) The carbapenemase gene-containing contig identified in step (ii) was aligned using the NCBI BLAST tool against the candidate plasmids obtained by PlasmidSeeker in step (i), only retaining candidate plasmids sharing ≥90% k-mers with the isolate as determined by PlasmidSeeker, and carbapenemase gene-containing contig BLAST alignment coverage of ≥90%; (iv) Finally, the specific CP-plasmid for each isolate was determined according to the following selection criteria consecutively: highest query coverage, highest k-mer coverage, and longest plasmid. For isolates that still had multiple valid candidate plasmid assignments, priority was given to the dominant plasmid that was determined for the given carbapenemase gene. Cluster assignment was derived from the cluster membership of the assigned carbapenemase-encoding plasmid. The largest cluster of carbapenemase-encoding plasmids for a given carbapenemase gene was termed as the dominant cluster.

Survival-Analysis Approach

We used the dataset shown in Appendix 2 Figures 8 and 9 comprising 98 donor-recipient pairs, we first classified each pair into one of 4 levels of taxonomic relatedness: same strain, same species, same genus, or different genera. The full dataset of log-conjugation frequencies for pKPC2 (522 datapoints) or pNDM1 (530 datapoints) was regressed against this variable. Unobserved plasmid conjugation events were taken to be left-censored, with the detection limit set at 1e-8 (as the minimum observed value was 5e-8). Donor-recipient pair was modeled as a random effect to account for unobserved heterogeneity specific to each pair (such as incompatible plasmids in the recipient).

Mathematically,

$$\log_{10} f = \beta_0 + \beta_1(\text{same species}) + \beta_2(\text{same genus}) + \beta_3(\text{other genus}),$$

where f is the conjugation frequency, β_0 is the baseline log-conjugation frequency between the same strain, and β_1, β_2, and β_3 denote the relative change in log-conjugation frequency for transfer between the same species, same genus, or other genus, respectively. The coefficients β_0, β_1, β_2, and β_3 were inferred using the survreg function of the R survival package (version 3.1-12) (*8*), with donor-recipient pair modeled as a gaussian frailty. The 95% confidence intervals were computed from 1,000 bootstrap replicates using the R boot package version 1.3-28 (*9*).

Data Availability

Raw sequence data from this study were uploaded to the NCBI Sequence Read Archive (SRA) Database under Bioproject accession numbers PRJNA757551 and PRJNA765801 for the Illumina short-read sequencing data, PRJNA801425 for the Oxford Nanopore Technologies (ONT) long-read sequencing data for the plasmid growth rate experiment, and PRJNA801415 for the ONT long-read sequencing data that contributed to the plasmid genome reference sequence database.

References

1. Seemann T. Prokka: rapid prokaryotic genome annotation. Bioinformatics. 2014;30:2068–9. PubMed https://doi.org/10.1093/bioinformatics/btu153

2. Murphy KC. Use of bacteriophage lambda recombination functions to promote gene replacement in *Escherichia coli*. J Bacteriol. 1998;180:2063–71. PubMed https://doi.org/10.1128/JB.180.8.2063-2071.1998

3. Choi KH, Mima T, Casart Y, Rholl D, Kumar A, Beacham IR, et al. Genetic tools for select-agent-compliant manipulation of *Burkholderia pseudomallei*. Appl Environ Microbiol. 2008;74:1064–75. PubMed https://doi.org/10.1128/AEM.02430-07

4. Datsenko KA, Wanner BL. One-step inactivation of chromosomal genes in *Escherichia coli* K-12 using PCR products. Proc Natl Acad Sci U S A. 2000;97:6640–5. PubMed https://doi.org/10.1073/pnas.120163297

5. Kvitko BH, Collmer A. Construction of *Pseudomonas syringae* pv. tomato DC3000 mutant and polymutant strains. Methods Mol Biol. 2011;712:109–28. PubMed https://doi.org/10.1007/978-1-61737-998-7_10

6. Chang AC, Cohen SN. Construction and characterization of amplifiable multicopy DNA cloning vehicles derived from the P15A cryptic miniplasmid. J Bacteriol. 1978;134:1141–56. PubMed https://doi.org/10.1128/jb.134.3.1141-1156.1978

7. Gupta SK, Padmanabhan BR, Diene SM, Lopez-Rojas R, Kempf M, Landraud L, et al. ARG-ANNOT, a new bioinformatic tool to discover antibiotic resistance genes in bacterial genomes. Antimicrob Agents Chemother. 2014;58:212–20. PubMed https://doi.org/10.1128/AAC.01310-13

8. Therneau T. A package for survival analysis in R [cited 2021 Aug 5]. https://github.com/therneau/survival

9. Canty A, Ripley BD. boot: Bootstrap R (S-Plus) functions [cited 2021 Aug 5]. https://cran.r-project.org/web/packages/boot/index.html

10. Blattner FR, Plunkett G III, Bloch CA, Perna NT, Burland V, Riley M, et al. The complete genome sequence of *Escherichia coli* K-12. Science. 1997;277:1453–62. PubMed https://doi.org/10.1126/science.277.5331.1453

11. Khetrapal V, Mehershahi K, Rafee S, Chen S, Lim CL, Chen SL. A set of powerful negative selection systems for unmodified *Enterobacteriaceae*. Nucleic Acids Res. 2015;43:e83. PubMed https://doi.org/10.1093/nar/gkv248

12. Mulvey MA, Schilling JD, Hultgren SJ. Establishment of a persistent *Escherichia coli* reservoir during the acute phase of a bladder infection. Infect Immun. 2001;69:4572–9. PubMed https://doi.org/10.1128/IAI.69.7.4572-4579.2001

13. Massip C, Branchu P, Bossuet-Greif N, Chagneau CV, Gaillard D, Martin P, et al. Deciphering the interplay between the genotoxic and probiotic activities of *Escherichia coli* Nissle 1917. PLoS Pathog. 2019;15:e1008029. PubMed https://doi.org/10.1371/journal.ppat.1008029

14. de Lorenzo V, Cases I, Herrero M, Timmis KN. Early and late responses of TOL promoters to pathway inducers: identification of postexponential promoters in *Pseudomonas putida* with lacZ-tet bicistronic reporters. J Bacteriol. 1993;175:6902–7. PubMed https://doi.org/10.1128/jb.175.21.6902-6907.1993

15. Lee IR, Molton JS, Wyres KL, Gorrie C, Wong J, Hoh CH, et al. Differential host susceptibility and bacterial virulence factors driving *Klebsiella* liver abscess in an ethnically diverse population. Sci Rep. 2016;6:29316. PubMed https://doi.org/10.1038/srep29316

16. Lee KW, Arumugam K, Purbojati RW, Tay QX, Williams RB, Kjelleberg S, et al. Draft genome sequence of *Klebsiella pneumoniae* strain KP-1. Genome Announc. 2013;1:e01082-13. PubMed https://doi.org/10.1128/genomeA.01082-13

17. Lam MMC, Wyres KL, Duchêne S, Wick RR, Judd LM, Gan YH, et al. Population genomics of hypervirulent *Klebsiella pneumoniae* clonal-group 23 reveals early emergence and rapid global dissemination. Nat Commun. 2018;9:2703. PubMed https://doi.org/10.1038/s41467-018-05114-7

18. Tan YH, Chen Y, Chu WHW, Sham LT, Gan YH. Cell envelope defects of different capsule-null mutants in K1 hypervirulent *Klebsiella pneumoniae* can affect bacterial pathogenesis. Mol Microbiol. 2020;113:889–905. PubMed https://doi.org/10.1111/mmi.14447

19. Chen Y, Marimuthu K, Teo J, Venkatachalam I, Cherng BPZ, De Wang L, et al. Acquisition of plasmid with carbapenem-resistance gene bla_{KPC2} in hypervirulent *Klebsiella pneumoniae*, Singapore. Emerg Infect Dis. 2020;26:549–59. PubMed https://doi.org/10.3201/eid2603.191230

20. Cook TB, Rand JM, Nurani W, Courtney DK, Liu SA, Pfleger BF. Genetic tools for reliable gene expression and recombineering in *Pseudomonas putida*. J Ind Microbiol Biotechnol. 2018;45:517–27. PubMed https://doi.org/10.1007/s10295-017-2001-5

Appendix 2 Table 1. Description of *Enterobacterales* strains used for carbapenemase-encoding plasmids in clinical isolates and hypervirulent *Klebsiella pneumoniae*, Singapore*

Strains	Features	Virulence factors	Source or reference
Enterobacter cloacae			
ATCC 13047	Type strain	ND	ATCC
Escherichia coli			
MG1655	K-12	ND	(*10*)
SLC-568	MG1655 *hsdS*::kan	ND	(*11*)
UTI89	Uropathogenic strain	ND	(*12*)
Nissle 1917	Probiotic strain	ND	(*13*)
S17–1 λpir	Conjugation donor carrying an integrated RP4–2 and λpir, TpR	ND	(*14*)
Enterobacter hormaechei			
ATCC 700323	Quality control strain	ND	ATCC
Klebsiella quasipneumoniae			
TTSH04	KL114, ST2037	ND	(*15*)
K. oxytoca			
8071169380	Clinical isolate	ND	This study
8071167205	Clinical isolate	ND	This study
K. variicola			
NUH59	KL10, ST906	ND	This study
K. pneumoniae			
ATCC 13883	KL3, ST3, type strain	ND	ATCC
NUH29	KL28, ST20	ND	(*15*)
KP-1	KL54, ST29, environmental isolate	ND	(*16*)
NUH03	KL2, ST65	ICEKp10, *iuc, clb, iro, rmpA*	(*15*)
NUH04	KL2, ST2039	ICEKp10, *iuc, clb, iro, rmpA*	(*15*)
NUH06	KL1, ST23	ICEKp3, *iuc, iro, rmpA*	(*15*)
NUH13	KL1, ST2044	ICEKp10, *iuc, clb, iro, rmpA*	(*15*)
NUH14	KL2, ST380	*iuc, clb, iro, rmpA*	(*15*)
NUH26	KL5, ST1049	ICEKp6, *iuc, iro, rmpA*	(*15*)
NUH40	KL5, ST1049	ICEKp6, *iuc, iro, rmpA*	This study
NUH61	KL2, ST86	*iuc, iro, rmpA*	This study
NUH66	KL2, ST2039	ICEKp10, *iuc, clb, iro, rmpA*	This study
NUH66Δ*wcaJ*	NUH66, *wcaJ* deletion (capsule null; deletion of initial glycosyltransferase)	*iuc, clb, iro, rmpA*	This study
SGH07	KL5, ST60	ICEKp1, *iro, rmpA*	(*15*)
SGH07Δ*wcaJ*	SGH07, *wcaJ* deletion	*iro, rmpA*	This study
TTSH21	KL5, ST60	ICEKp1, *iro, rmpA*	This study
TTSH25	KL2, ST2039	ICEKp10, *iuc, clb, iro, rmpA*	(*15*)
TTSH25Δ*wcaJ*	TTSH25, *wcaJ* deletion (capsule null; deletion of initial glycosyltransferase)	*iuc, clb, iro, rmpA*	This study
SGH10	KL1, ST23	ICEKp10, *iuc, clb, iro, rmpA*	(*17*)
SGH10Δ*wcaJ*	SGH10, *wcaJ* deletion (capsule null; deletion of initial glycosyltransferase)	ICEKp10, *iuc, clb, iro, rmpA*	(*18*)
SGH10Δ*rmpA*	SGH10, *rmpA* deletion (deletion of *rmpA* abrogates the hypermucoviscous phenotype)	ICEKp10, *iuc, clb, iro*	(*19*)
SGH10ΔGIE492	SGH10, GIE492 deletion (deletion of genomic island responsible for the production of microcin E492 and salmochelin)	ICEKp10, *iuc, clb, rmpA*	This study
SGH10ΔT6SS1 ΔT6SS3	SGH10, T6SS1 and T6SS3 deletion (deletion of the two clusters of type 6 secretion system)	ICEKp10, *iuc, clb, iro, rmpA*	This study
SGH10ΔICEKp10	SGH10, ICEKp10 deletion (deletion of the chromosomal integrative and conjugative element encoding colibactin and yersiniabactin)	*iuc, iro, rmpA*	This study
SGH10Δ*mrkA*	SGH10, *mrkA* deletion (deletion of the gene encoded for major subunit of type 3 fimbrae)	ICEKp10, *iuc, clb, iro, rmpA*	This study
SGH10ΔKpVP	SGH10, KpVP deletion	ICEKp10, *clb*	This study
SGH10Δ*mrkA*ΔKpVP	SGH10, KpVP and *mrkA* deletion	ICEKp10, *clb*	This study

*ATCC, American Type Culture Collection (https://www.atcc.org); ND, not done.

Appendix 2 Table 2. Description of carbapenemase-encoding plasmids used in a study of clinical *Enterobacterales* isolates and hypervirulent *Klebsiella pneumoniae*, Singapore

Plasmid	Features	Source
pACYC184	Low copy number plasmid with a p15A replicon, CmR, TcR	(*6*)
pFLPe2	Source of zeocin resistance gene and Flp recombinase gene driven by PrhaB promoter, ZnR	(*3*)
pK18mobsacB	Source of sucrose counter-selection gene *sacB*, KmR	(*5*)
pR6KmobsacB	Conditional replicative plasmid carrying *sacB* and oriT, KmR	(*18*)
pKD46	Source of Red recombinases driven by Pbad promoter, ApR	(*4*)
pACYC-flp-Red-sacB	A p15A plasmid carrying *Flp* gene, Lambda Red recombinase genes and *sacB* gene, TcR	This study
pKD3	Source of chloramphenicol resistance gene flanked by FRT sites (FRT-Cm), CmR	(*4*)
pKD4	Source of kanamycin resistance gene flanked by FRT sites (FRT-Km), KmR	(*4*)
pKPC2	Conjugative plasmid from ENT494 carrying a *bla*$_{KPC-2}$ gene, CbR	(*19*)
pKPC2^{KmR}	pKPC2 containing a kanamycin resistance gene from pKD4, CbR, KmR	This study
pKPC2^{KmR}Δ*higB*	pKPC2, *higB* was replaced with a kanamycin resistance gene, KmR	This study
pKPC2Δ*parB*	pKPC2, *parB* was replaced with a chloramphenicol resistance gene, CmR	This study
pNDM1	Conjugative plasmid from ENT448 carrying a *bla*$_{NDM-1}$ gene, CbR	This study
pNDM1^{KmR}	pNDM1 containing a kanamycin resistance gene from pKD4, CbR, KmR	This study
pRK2-AraE	IncP plasmid encoding *trfA* replication protein	(*20*)

Appendix 2 Table 3. Primers used in mutant generation for carbapenemase-encoding plasmids in clinical *Enterobacterales* isolates and hypervirulent *Klebsiella pneumoniae*, Singapore*

Name	Sequence
K2_wcaJ Up F	TATGACATGATTACGAATTCAGCTCTGGCTGGTCCACTTA
K2_wcaJ Up R	AGTTTTTCTCACATTTAAGCTGCGAACG
K2_wcaJ Down F	AAATGTGAGAAAAACTCTTAGTGTTGCCATGA
K2_wcaJ Down R	CTTGCATGCCTGCAGACGCGAGAATGGAATTGTTC
K2_wcaJ Check F	GGTTGAAAACGGAGACGGTA
K2_wcaJ Check R	CGTTGTTGCGAGTCATTCAG
K5_wcaJ Up F	TATGACATGATTACGAATTCTATCTGGAAGACTGGCACGA
K5_wcaJ Up_R	CGCGGAATGACATGCATAACCTCAAATCAATC
K5_wcaJ Down_F	TGCATGTCATTCCGCGTTGTTCATTTCT
K5_wcaJ Down_R	CTTGCATGCCTGCAGAGCTCTGGCTGGTCCACTTA
K5_wcaJ Check_F	TTTTAGTTTTGTAACCTATGGCAGTT
K5_wcaJ_Check_R	CCGATCTGTTGCTTGGACAT

*F, forward; R, reverse.

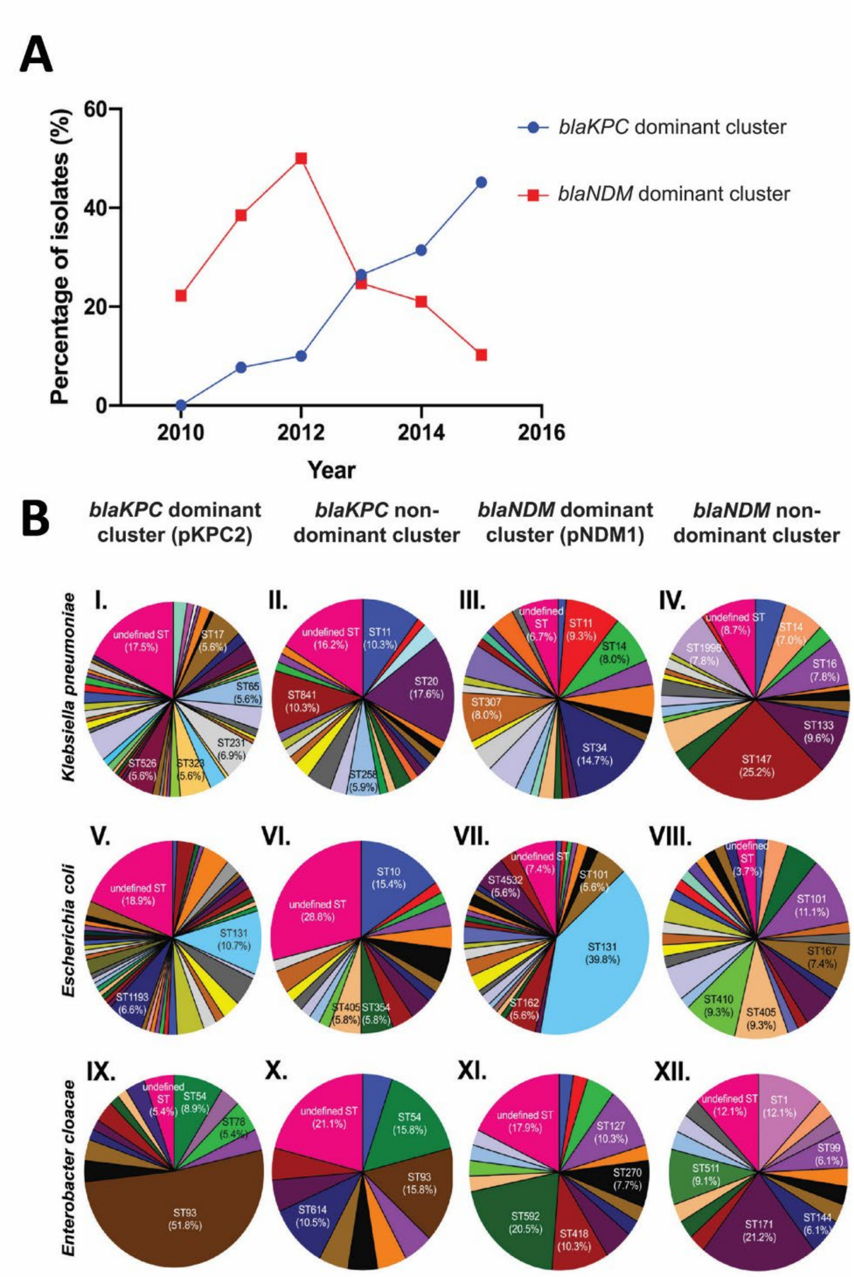

Appendix 2 Figure 1. Distribution of *bla*$_{KPC}$ and *bla*$_{NDM}$ plasmids in the Carbapenemase-Producing *Enterobacterales* in Singapore collection, 2010–2015. A) Percentage of isolates belonging to *bla*$_{KPC}$ dominant cluster or *bla*$_{NDM}$ dominant cluster in each year. B) Distribution of *bla*$_{KPC}$ dominant cluster (pKPC2), *bla*$_{KPC}$ nondominant cluster (other non-pKPC2 *bla*$_{KPC}$ plasmids), *bla*$_{NDM}$ dominant cluster (pNDM1), and *bla*$_{NDM}$ nondominant cluster (other non-pNDM1 *bla*$_{NDM}$ plasmids) in numerous ST sequence types of *Klebsiella pneumoniae* strains (I–IV), *Escherichia coli* (V–VIII), and *Enterobacter cloacae* (IX–XII). Each slice in pie chart indicates 1 unique ST. Undefined STs indicate strains that could not be assigned to an ST. ST, sequence type.

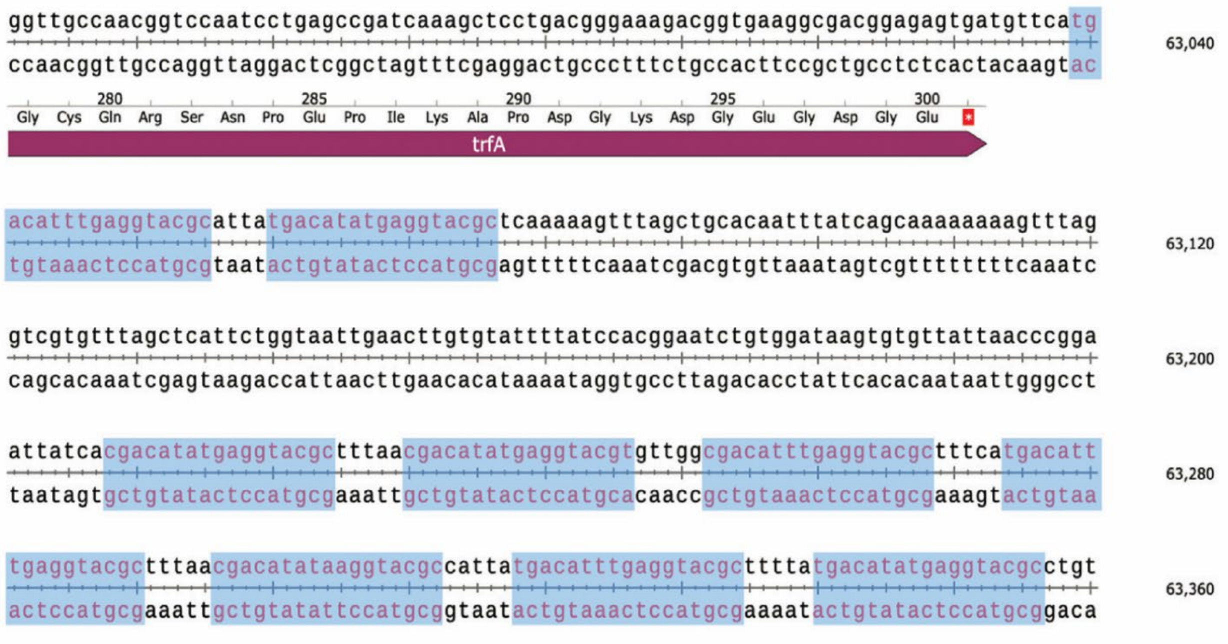

Appendix 2 Figure 2. Downstream *trfA* gene sequence detected in carbapenemase-encoding plasmids in clinical *Enterobacterales* isolates and hypervirulent *Klebsiella pneumoniae*, Singapore. The *trfA* gene was detected in pKPC2 containing 9 17-bp repeats (blue highlighting) with similarity to the 5 17-bp repeats in the pRK2 oriV sequence. The 17-bp repeats of $^{T}/_{C}$GACAT$^{A}/_{T}$T$^{G}/_{A}$AGGTACG$^{C}/_{T}$ were detected downstream of *trfA* gene, which resembled the pRK2 oriV repeats of TGACA$^{C}/_{A}$$^{T}/_{G}$TGAGGGGC$^{A}/_{G}$$^{G}/_{C}$. Image was generated by using SnapGene (https://www.snapgene.com).

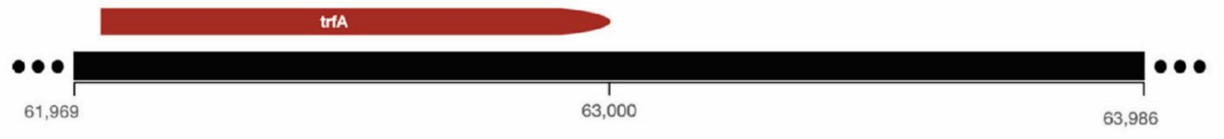

Appendix 2 Figure 3. Replicative pKPC2 fragment detected in carbapenemase-encoding plasmids in clinical *Enterobacterales* isolates and hypervirulent *Klebsiella pneumoniae*, Singapore. The *trfA* gene and the iteron (a total of 2,018 bp) was cloned by PCR by using forward primer TAAGCAGGATCCGATTACATTGCTGAGAATA TCCAGTATTTAAA TAC; and reverse primer TGCTTAGGATCCAATTTAGAACATTCGAAAAATGATCCAA TTTCGCAT and then inserted into pR6K after *Bam*HI digestion. The fusion plasmid was transformed into *Escherichia coli* Stellar–competent cells. The insertion sequence was verified by Sanger sequencing by using isolated plasmids from successfully transformed cells.

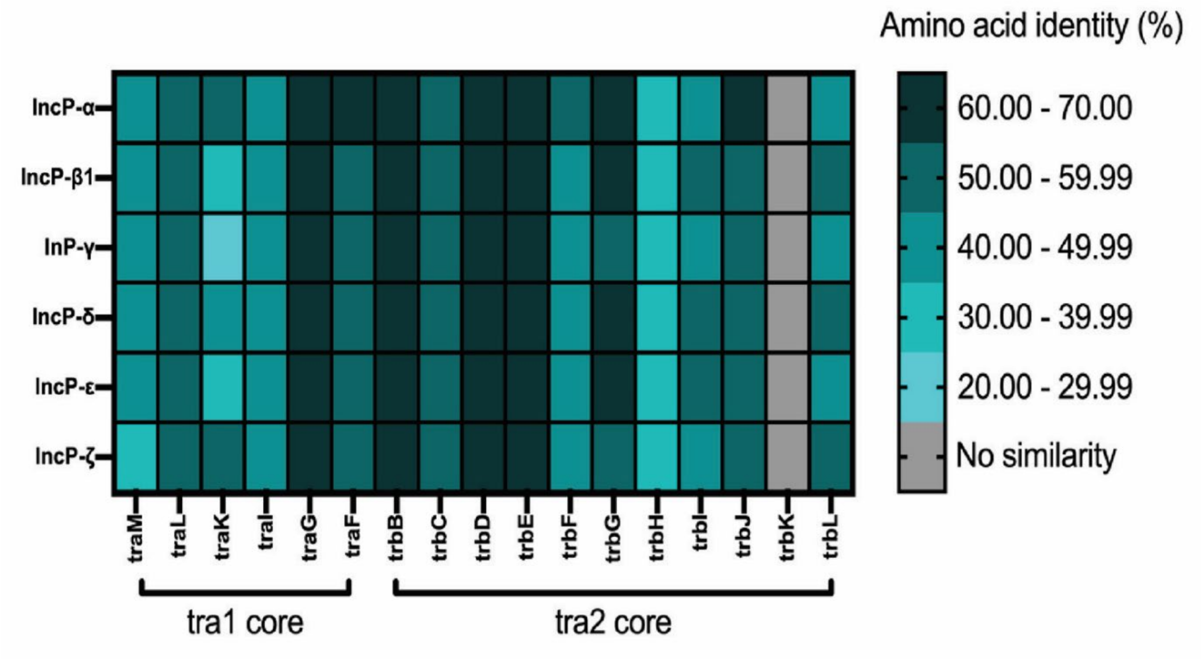

Appendix 2 Figure 4. Heatmap comparing the conjugative genes of pKPC2 to the distinct conjugative genes from IncP in carbapenemase-encoding plasmids from clinical *Enterobacterales* isolates and hypervirulent *Klebsiella pneumoniae*, Singapore. The protein sequence of the pKPC2 conjugative genes was compared with the IncP plasmids' conjugative genes (*tra1* and *tra2* cores) by using BlastP (https://blast.ncbi.nlm.nih.gov). The heatmap shows amino acid percent identity from each comparison.

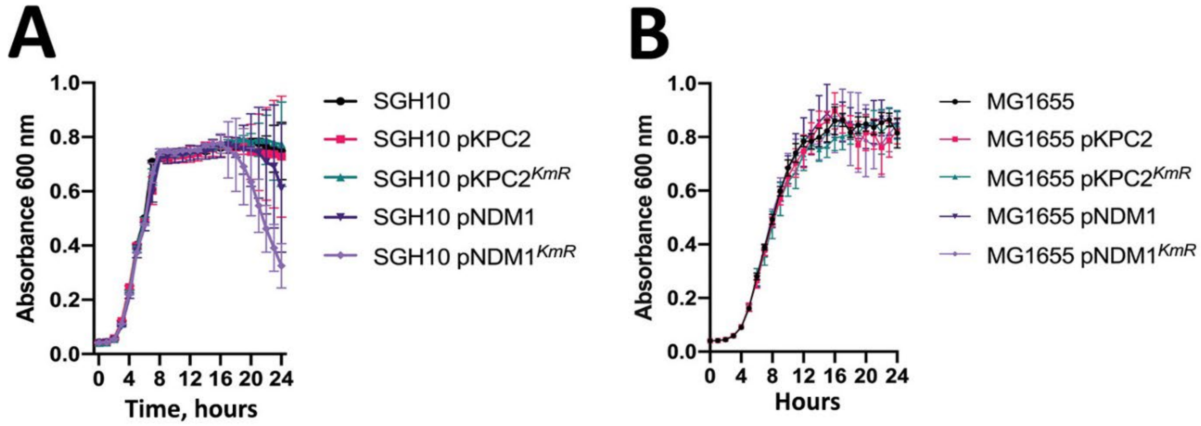

Appendix 2 Figure 5. Growth curve of carbapenemase-encoding plasmids in clinical *Enterobacterales* isolates and hypervirulent *Klebsiella pneumoniae*, Singapore. A) Representative growth curve of *K. pneumoniae* SGH10. B) Representative growth curve of *E. coli* MG1655. SGH10 and MG1655 were grown with or without plasmids (pKPC2, pKPC2^{KmR}, pNDM1, pNDM1^{KmR}) in M9 media supplemented with 0.4% glucose and 0.4% casamino acids for 24 hours at 37°C. Symbols indicate mean; error bars denote standard deviation.

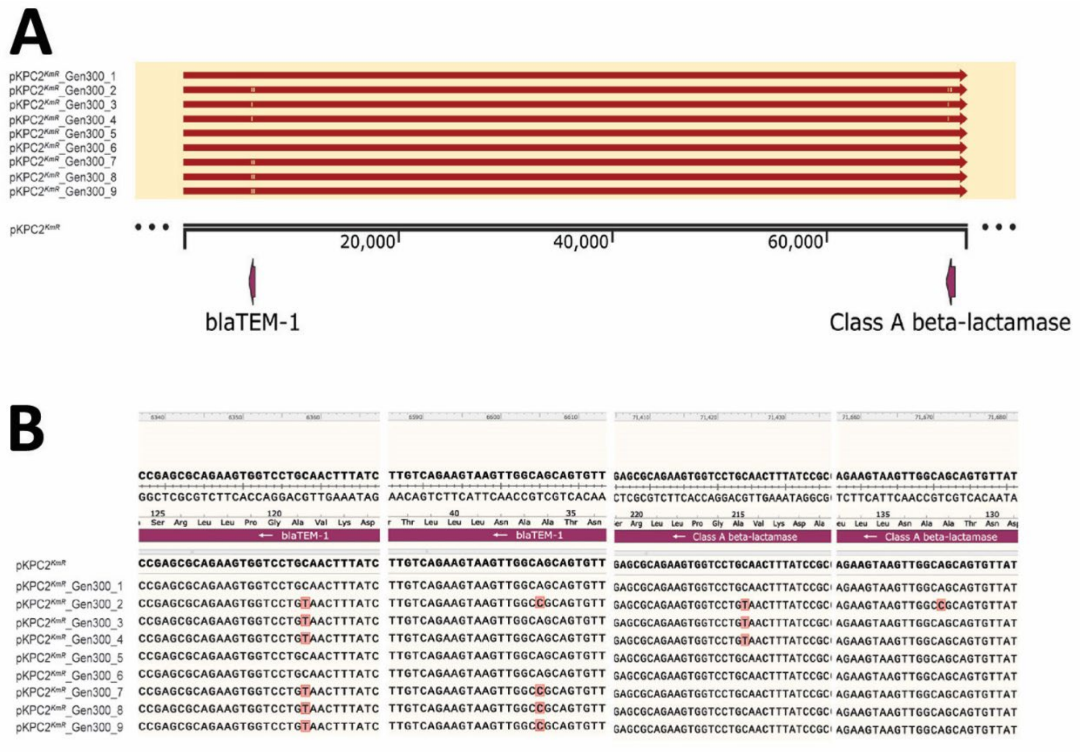

Appendix 2 Figure 6. Sequence comparison between carbapenemase-encoding plasmids in clinical *Enterobacterales* isolates and hypervirulent *Klebsiella pneumoniae*, Singapore. Multiple pKPC2^{KmR} were isolated from *K. pneumoniae* SGH10 grown over 300 generations (pKPC2^{KmR}_Gen300) and compared with the original pKPC2^{KmR}. A) Sequence alignments of pKPC2^{KmR} and pKPC2^{KmR}_Gen300. White gaps on the red bars indicate single nucleotide mismatch. B) Identified nucleotide mismatches over multiple comparisons. Red highlights indicate positions of the nucleotide mismatch identified in pKPC2^{KmR}_Gen300.

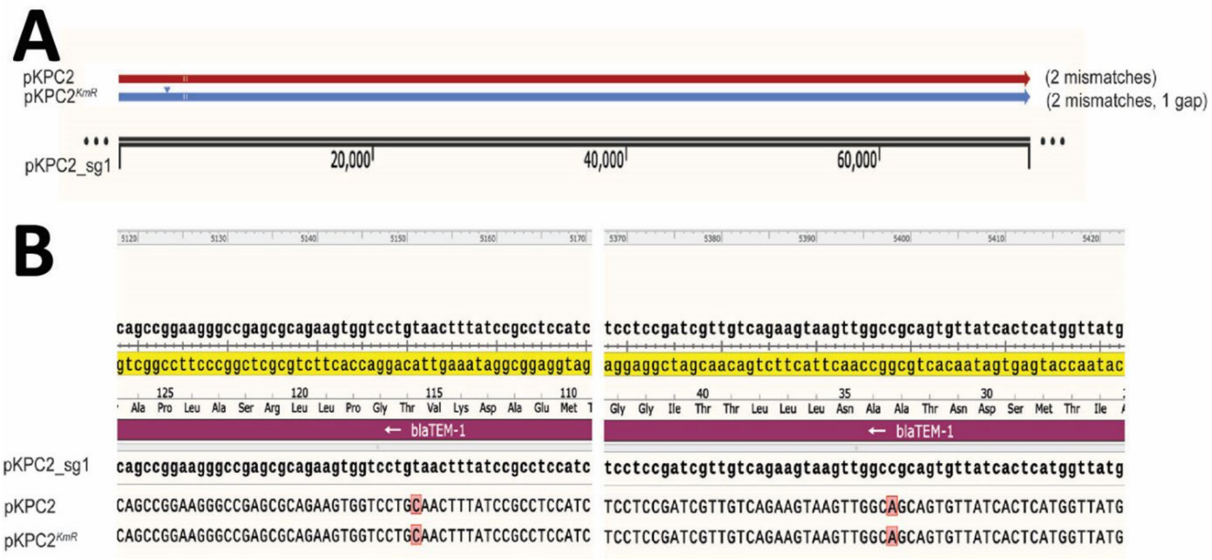

Appendix 2 Figure 7. Sequence comparison between pKPC2, pKPC2^{KmR}, and pKPC2_sg1 (GenBank accession no. MN542377) detected in carbapenemase-encoding plasmids in clinical *Enterobacterales* isolates and hypervirulent *Klebsiella pneumoniae*, Singapore. A) Comparison between the pKPC2 and pKPC2^{KmR} plasmids from this study with the pKPC2_sg1 plasmid from clinical isolate ENT494. Plasmid alignment showed 2 mismatches (white lines on the red and blue bars) on *bla*$_{TEM-1}$ gene of pKPC2 and pKPC2^{KmR} compared with pKPC2_sg1. One gap (indicated by the blue arrowhead) was identified on pKPC2^{KmR} corresponding to the kanamycin gene insertion. B) Nucleotide sequence of the 3 plasmids showing the position of the mismatches (T>C at position 5151 and C>A at position 5398 of the pKPC2_sg1 sequence). Both mismatches were on *bla*$_{TEM-1}$ gene.

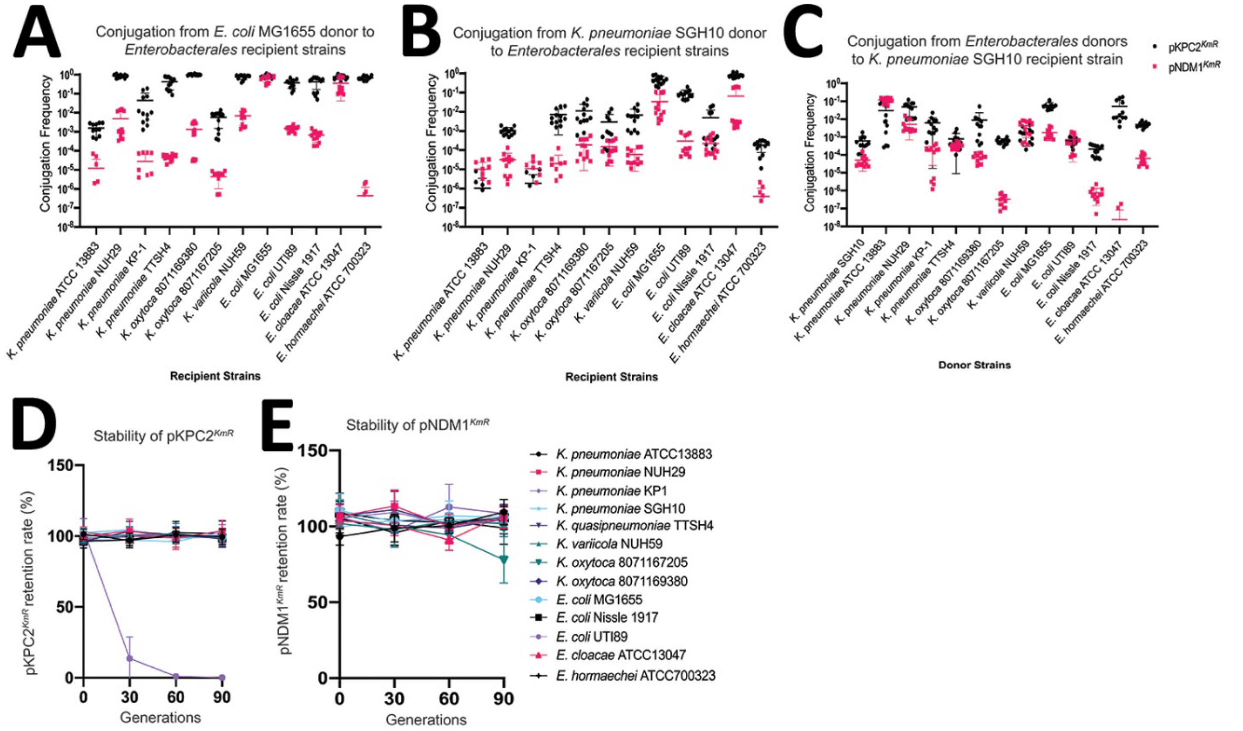

Appendix 2 Figure 8. Conjugation frequency and stability of pKPC2^{KmR} and pNDM1^{KmR} among various clinical *Enterobacterales* isolates and hypervirulent *Klebsiella pneumoniae*, Singapore. A–C) Conjugation frequency of pKPC2^{KmR} and pNDM1^{KmR} from *E. coli* MG1655 donor strain (A) and from *K. pneumoniae* SGH10 donor strain (B) to a panel of *Enterobacterales* recipient strains; and C) from the panel of *Enterobacterales* donor strains to *K. pneumoniae* SGH10 recipient strain. Each symbol represents 1 experimental replicate with a total of 12 replicates. Data points that are not seen on the graphs indicate no detectable transconjugant. D,E) Stability of pKPC2^{KmR} (D) and pNDM1^{KmR} (E) in various *Enterobacterales* strains grown in lysogeny broth up till generation 90. Symbols indicate mean; error bars indicate SD from 3 independent experiments.

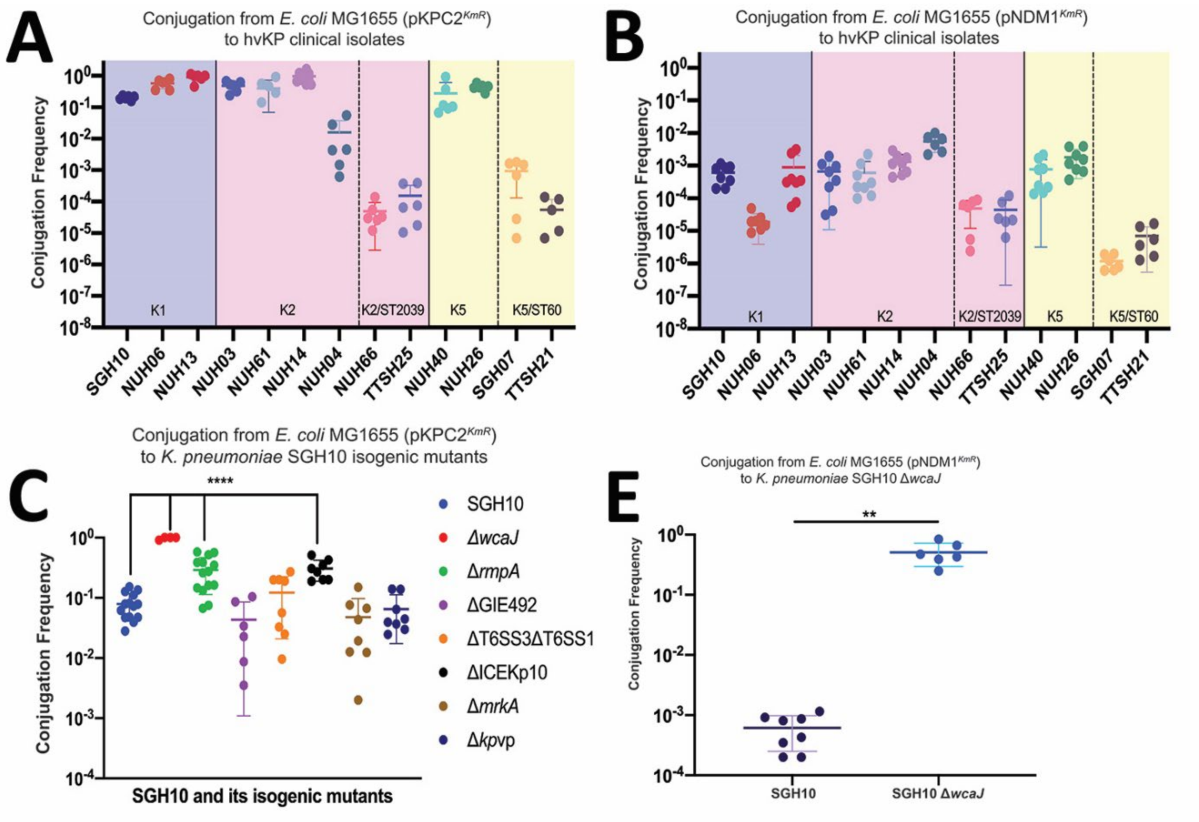

Appendix 2 Figure 9. Conjugation frequency of pKPC2^{KmR} and pNDM1^{KmR} to hypervirulent *Klebsiella pneumoniae* clinical isolates in a study of dominant carbapenemase-encoding plasmids in clinical *Enterobacterales* isolates, Singapore. A,B) Conjugation frequency of pKPC2^{KmR} (A) or pNDM1^{KmR} (B) from *Escherichia coli* MG1655 donor to hypervirulent *K. pneumoniae* clinical isolates. C) Conjugation frequency of pKPC2^{KmR} from *E. coli* MG1655 donor to *K. pneumoniae* SGH10 and its isogenic mutants (Appendix 2 Table 1) based on 1-way ANOVA statistical analysis. D) Conjugation frequency of pNDM1^{KmR} from *E. coli* MG1655 donor to *K. pneumoniae* SGH10 and its capsule null mutant (Δ*wcaJ*) based on unpaired Student *t*-test. Error bars indicate SD from 3 independent experiments. **p<0.01; ****p<0.0001.

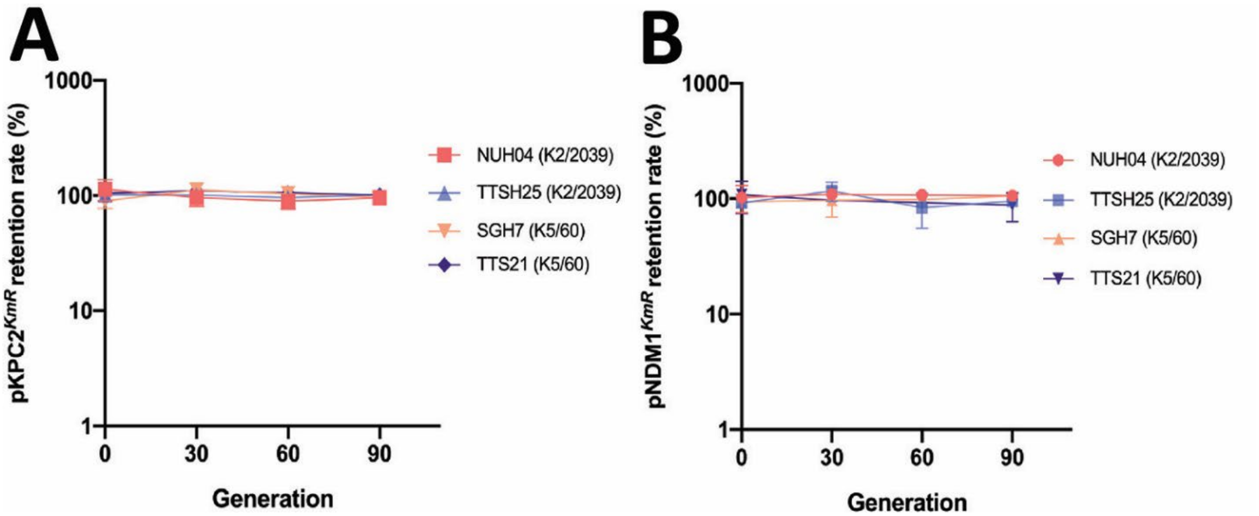

Appendix 2 Figure 10. Plasmid stability in low conjugating hypervirulent *Klebsiella pneumoniae* strains in a study of carbapenemase-encoding plasmids in clinical *Enterobacterales* isolates and hypervirulent *Klebsiella pneumoniae*, Singapore. A,B) Stability of pKPC2^{KmR} (A) or pNDM1^{KmR} (B) in low conjugating hypervirulent *K. pneumoniae* A-KLASS isolates via continuous culture in lysogeny broth for 90 generations. Error bars indicate mean +SD from 3 independent experiments.

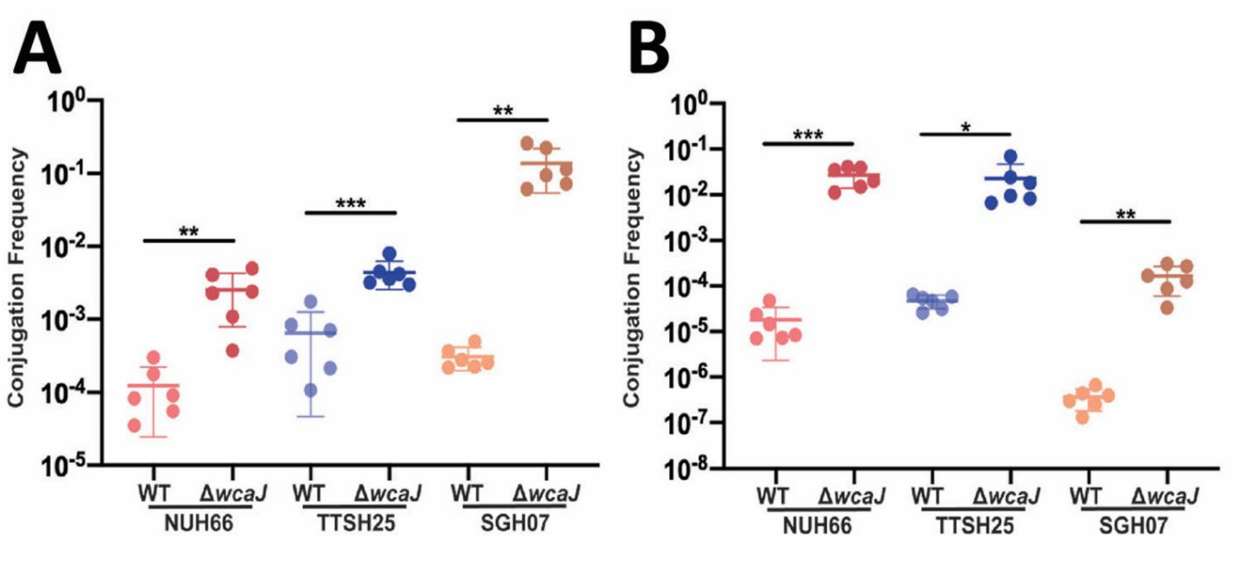

Appendix 2 Figure 11. Conjugation frequency of carbapenemase-encoding plasmids in clinical *Enterobacterales* isolates and hypervirulent *Klebsiella pneumoniae*, Singapore. A) Conjugation of pKPC2^{KmR}, pKPC2^{KmR}, and pNDM1^{KmR} to capsule null mutant of low conjugating hypervirulent *K. pneumoniae* strains. B) Conjugation of pNDM1^{KmR} from *Escherichia coli* MG1655 to NUH66, TTSH25, or SGH07 wildtype strains and capsule null mutant (Δ*wcaJ*). Error bars indicate mean +SD from 3 independent experiments. *p<0.05; **p<0.01; ***p<0.001.
